# Contrasting trophic transfer patterns of cadmium and mercury in the Arctic marine food web of east Hudson Bay, Canada

**DOI:** 10.1007/s11356-024-32268-3

**Published:** 2024-02-20

**Authors:** Jillian Rohonczy, John Chételat, Stacey A. Robinson, Lucassie Arragutainaq, Joel P. Heath, Christine McClelland, Raymond Mickpegak, Mark R. Forbes

**Affiliations:** 1 Department of Biology, Carleton University, Ottawa, ON K1S 5B6 Canada; 2 Environment and Climate Change Canada, National Wildlife Research Centre, Ottawa, ON K1A 0H3 Canada; 3 Sanikiluaq Hunters and Trappers Association, Sanikiluaq, NU XAO 0W0 Canada; 4 Arctic Eider Society, Sanikiluaq, NU X0A 0W0 Canada; 5 Sakkuq Landholding Corporation, Kuujjuarapik, QC J0M 1G0 Canada

**Keywords:** Heavy metals, Bioaccumulation, Stable isotopes, Methylmercury, Subarctic, Biomagnification, Food web

## Abstract

We investigated trophic transfer of cadmium (Cd) through an Arctic marine food web in Hudson Bay and compared it with mercury (Hg), a metal known to strongly biomagnify. We evaluated blue mussel, sea urchin, common eider, sculpin, Arctic cod, and ringed seal for the influence of dietary and biological variables on variation in Cd and Hg concentrations. Age and size influenced metal concentrations among individuals within a vertebrate species. Consumer carbon and sulfur isotope values were correlated with their Cd and Hg concentrations, indicating habitat-specific feeding influenced metal bioaccumulation. Trophic transfer patterns for Cd depended on the vertebrate tissue, with food web biodilution observed for the muscle but not the liver. Liver Cd concentrations were higher in ringed seal and some common eider relative to prey. In contrast, we observed mercury biomagnification for both tissues. Tissue- and species-specific physiology can explain discrepancies of Cd trophic transfer in this Arctic marine food web.

**Supplementary Information:**

The online version contains supplementary material available at 10.1007/s11356-024-32268-3.

## Introduction

Cadmium (Cd) is a non-essential element present in marine food webs. Exposure to elevated levels of Cd can result in health consequences for fish and wildlife. For example, dietary Cd exposure has been linked to kidney, bone, and reproductive damage in mammals (summarized in Marettová et al. 2015; Thévenod and Lee 2013) and has immunotoxic effects (Desforges et al. 2016). Additionally, exposure to higher concentrations of Cd can reduce fish growth (Ng and Wood 2008), reduce nutrition uptake, and cause kidney and reproductive organ damage in birds (Marettová et al. 2015; Wayland and Scheuhammer 2011). Researchers have detected elevated concentrations of Cd in tissues of marine mammals and seabirds in Arctic environments (Brown et al. 2016; Mallory et al. 2014; Wayland et al. 2001). Ocean food web studies have typically concluded that Cd does not biomagnify (Campbell et al. 2005; Cardwell et al. 2013; Gao et al. 2021; Gray 2002). However, uncertainties remain regarding Cd transfer in marine food webs since Cd biomagnification has been observed in benthic food webs with macroinvertebrates (Cheung and Wang 2008; Croteau et al. 2005) and since Cd concentrations were found to increase with trophic position in Arctic seabirds (Øverjordet et al. 2015).

Both natural and anthropogenic sources of Cd contribute to exposure levels in the Arctic. Cadmium enters the Arctic Ocean via long-range atmospheric transport and via deposition of volcanic and industrial emissions (De Vera et al. 2021), though the fluxes are relatively low compared to background transport via oceanic currents (Macdonald et al. 2000). Natural and anthropogenic sources of Cd also contribute to coastal environments via river transport of Cd-containing materials (Lambelet et al. 2013), including from mining (Søndergaard and Mosbech 2022). In addition, climate change-induced alterations to the Arctic environment, including thawing of permafrost, loss of costal sea ice, and atmospheric changes are leading to changes in transport and deposition of metals in Arctic aquatic ecosystems (AMAP 2021).

Carbon, nitrogen, and sulfur isotopes (δ^13^C, δ^15^N, and δ^34^S) are widely used to evaluate the role of trophic position and habitat-specific feeding on metal bioaccumulation. Dietary δ^13^C values undergo minimal change as carbon is transferred through the food web; therefore, δ^13^C values can provide information on relative carbon sources (Post 2002). In aquatic ecosystems, higher δ^13^C values indicate that individuals are feeding on benthic carbon sources, and lower (more negative) δ^13^C values indicate that individuals are feeding on pelagic carbon sources (Post 2002). Similarly, sulfur stable isotopes can provide information on habitat-specific feeding of biota. The δ^34^S values of pelagic-feeding Arctic marine mammals are higher than benthic-feeding Arctic marine mammals (Szpak and Buckley 2020). Sulfur stable isotopes can also indicate feeding in coastal estuarine habitats due to lower values in biota influenced by freshwater inputs of sulfate (Fry 2013). Finally, variation in δ^34^S values can reflect differences in the degree of sulfate reduction in foraging habitats (Habicht and Canfield 1997). The δ^15^N values of biota provide information on the trophic position of individuals as δ^15^N values increase significantly between a consumer and their food source (Post 2002).

The purpose of this study was to determine the role of food web processes in controlling high Cd concentrations detected in Arctic marine biota. We sampled animals from multiple trophic levels (primary, secondary, and tertiary consumers) and from both benthic and pelagic habitats near four communities in east Hudson Bay (EHB), Canada. We also explored the role of biological variables such as age and size in explaining variation in Cd concentrations within and between sampled species from EHB. Further, we compared the dynamics of Cd in the EHB food web to those of mercury (Hg). In aquatic systems, microbial and photochemical processes can transform inorganic Hg into an organic form called methylmercury (MeHg) (Lehnherr 2014). Methylmercury is well studied in aquatic food webs and is known to strongly biomagnify in Arctic marine food webs (Braune et al. 2015). Therefore, if Cd does biomagnify in the Hudson Bay food web, we would expect to see similar trends to those of Hg in the same food web. This research addresses uncertainties regarding processes that elevate Cd concentrations in Arctic marine vertebrates feeding on benthic and pelagic prey.

## Methods

### Study area

**Fig. 1:**
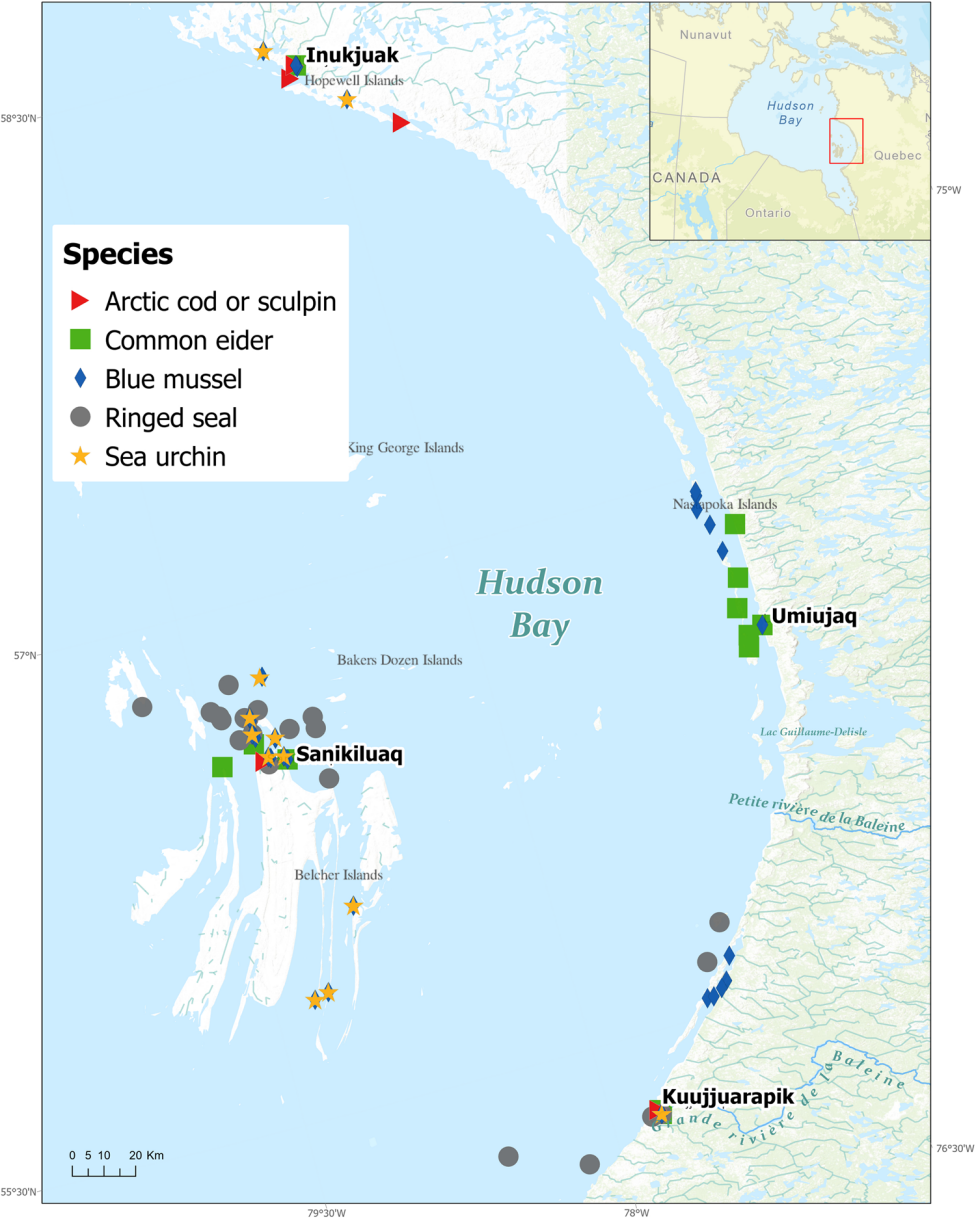
Map of eastern Hudson Bay (Quebec and Nunavut, Canada) illustrating the locations where marine biota were collected in the study area. Location data were not available for all samples, and in those cases, the nearest community was used to indicate collection site

Hudson Bay is a Canadian subarctic marine ecoregion and the most southerly extension of inland sea with Arctic oceanic conditions (Gupta et al. 2022; Stewart and Barber 2010). The ice cover period for Hudson Bay extends from approximately December to June in the southern reaches (Gupta et al. 2022). This ecosystem supports a typical Arctic marine food web that includes keystone species such as Arctic cod (*Boreogadus saida*), ringed seal (*Pusa hispida*), and polar bear (*Ursus maritimus*) (Stewart and Barber 2010). The study area in EHB encompassed three communities along the coast (Kuujjuaraapik, Umiujaq, and Inukjuak) and a fourth community (Sanikiluaq) on the Belcher Islands, within a latitudinal range of 55° 17′ N to 58° 27′ N (Fig. 1). Little anthropogenic development has occurred along the shores in the study area, but Hudson Bay receives large inputs of fresh water from its watershed, including from major hydroelectric developments in northern Quebec and Manitoba (Stewart and Barber 2010).

Several bioindicator taxa were selected to design a study that incorporated both benthic and pelagic trophic transfer in the marine food web. Ringed seal in Hudson Bay feed on forage fish such as species of sand lance (*Ammodytes* spp.) and capelin (*Mallotus villosus*), benthic sculpin (Cottidae), and to a lesser extent Arctic cod, and crustacean invertebrates such as amphipods and mysids (Chambellant et al. 2013). Common eiders (*Somateria mollissima*) are diving seaducks that consume benthic invertebrates such as molluscs, crustaceans, and polychaetes in coastal areas (Galaktionov et al. 2021). Arctic cod and sculpin occupy pelagic and benthic habitats, respectively; however, both fishes consume a mix of pelagic and benthic fauna (Buckley and Whitehouse 2017; Landry et al. 2018). Finally, blue mussel (*Mytilus edulis*) and sea urchin (Echinoidea) were selected as pelagic and benthic bioindicators, respectively, due to filter feeding of mussels on particulate organic matter in the water column and feeding on benthic algae and on plants by sea urchins.

### Sample collection

Wildlife and marine invertebrate samples were collected through community-based projects during the summer, fall, and winter between the years of 2014 and 2017 by Inuit hunters from the four communities. We provided sampling kits and field sheets to record collection information by local hunters who harvested common eiders and ringed seals. In most cases, the liver and muscle were removed in the field and placed in the sampling bags from the kits for storage and transport, although a few whole eiders were shipped frozen to the National Wildlife Research Centre (NWRC), a facility of Environment and Climate Change Canada in Ottawa, Canada, for dissection. Ringed seal length and girth were recorded in field sheets. Arctic cod and sculpin were line caught and shipped in plastic bags frozen whole. Blue mussel and sea urchin were collected by hand or by using a bottom-dredge net, usually in groups of ten animals per site (range 4–11), and frozen for storage and transport. Zooplankton were collected only at Sanikiluaq using a 1 m diameter, 1000-µm mesh net, towed by boat, and frozen in 500-mL HDPE containers for shipment.

In the laboratory at NWRC, blue mussel soft tissues and sea urchin gonads were removed from shells and processed as pools. Fish were weighed, measured for total length, and dissected to retrieve the liver and dorsal muscle. Otoliths were collected and aged using the crack and burn method by AAE Tech (La Salle, Manitoba, Canada). The fish stomachs were examined, and if present, undigested prey was collected for analysis. Muscle samples, fish livers, and small prey samples were freeze-dried for 48 h and manually homogenized within their containers. Seal and eider livers, mussel soft tissues, urchin gonads, and the larger prey samples were mechanically homogenized and freeze-dried for 48 h.

### Laboratory analyses

All chemical analyses were performed on freeze-dried and homogenized material, and concentrations are reported on a dry weight basis. Total mercury (THg) concentrations were measured in tissues (*n* = 436) using a Direct Mercury Analyzer 80 (Milestone Inc., Shelton, Connecticut, USA) at NWRC. One replicate was analyzed for every ten samples and had a mean relative standard deviation of 2.5% (*n* = 52). Six types of reference materials were analyzed with a mean recovery of 95% (range 84–108%, *n* = 108) (Table S1). The method detection limit was 0.007 µg/g dry weight or lower, and samples were always above detection.

Methylmercury (MeHg) is the chemical form of Hg that biomagnifies through food webs, and invertebrate tissues and liver can have variable proportions of THg as MeHg, while the muscle contains predominately MeHg (Chételat et al. 2020). A subset of zooplankton, prey items, blue mussel, sea urchin, fish liver, and seal liver samples (*n* = 96) was analyzed for MeHg at NWRC, Flett Research Ltd. (Winnipeg, Canada), or the University of Montreal (Montreal, Canada), depending on the year. Duplicate samples were analyzed and had a mean relative standard deviation of 2.9% (*n* = 13). The mean recovery of certified reference materials was 92% (range 84–104%, *n* = 15) (Table S2). The method detection limit was 0.004 µg/g dry weight or lower.

Cadmium concentrations were analyzed for the full sample set (*n* = 345) by acid digestion and detection on an inductively coupled plasma mass spectrometer (ICP-MS) at NWRC or RPC laboratories (Fredericton, Canada). Duplicate samples were analyzed and had a mean relative standard deviation of 2.1% (*n* = 14). The mean recovery of certified reference materials was 98% (range 93–124%, *n* = 18) (Table S3). The method detection limit was 0.01 µg/g dry weight or lower.

Stable isotope ratios of carbon, nitrogen, and sulfur in biota were measured in invertebrates and vertebrate muscle (*n* = 271) on a DeltaPlus XP Isotope Ratio Mass Spectrometer interface by a ConFlo II to a Vario EL III elemental analyzer at the Ján Veizer Stable Isotope Laboratory at the University of Ottawa (Ottawa, Canada). Lipid content was measured in the livers of cod and sculpin and a subset of ringed seal liver, eider liver, blue mussel, and sea urchin (*n* = 124). Acid hydrolysis method AOAC 948.15 was employed to quantify tissue lipid content at RPC laboratories.

### Statistical analyses

All statistical analyses were completed using R version 4.0.2 (R Core Team 2021). Information regarding testing of assumptions for statistical tests can be found in Appendix A—Supplementary Information. The complete dataset for this study is publicly available on the Open Data Portal of the Government of Canada (GOC 2022).

Sculpin and Arctic cod had variable and in some cases high lipid content in their livers, which dilute protein-bound metal concentrations (Amlund et al. 2007; López-García et al. 2014). For Arctic cod, for example, there was a negative correlation between lipid content and metal concentrations in the liver (Table S4). Therefore, we used lipid-normalized concentrations for the liver of fish, eider, and seal and whole-body measurements of blue mussel and gonads of sea urchin in the statistical models using the liver. Lipid normalization was calculated as follows: lipid normalized [metal] = [metal]/(1 − lipid portion), where lipid portion has values between 0 and 1 and is assumed to contain negligible metal concentrations (Amlund et al. 2007; López-García et al. 2014). Normalization was applied using individual % lipid values for Arctic cod and sculpin liver, which were highly variable, and average % lipid values for eider liver, seal liver, mussel, and urchin, which had low and consistent lipid content. Vertebrate muscle is typically low in lipid (≤ 10%) (Braune et al. 1999; Pedro et al. 2019; Schantz et al. 1993) and similar to lipid content of blue mussel and sea urchin (8% and 21%, respectively); therefore, statistical models that included the muscle for higher trophic levels did not include lipid-normalized data.

We used THg concentrations for the liver and for the muscle and MeHg concentrations for invertebrates. The % MeHg of the muscle exceeds 80% for included species (Dietz et al. 1996; Houserová et al. 2007; Wagemann et al. 1998). However, the % MeHg of invertebrate tissues is more variable, and we wanted to account for the greater variation. Therefore, we only included the subset of invertebrates that had MeHg concentrations measured in our analyses. The % MeHg in the liver of vertebrates is more variable (50–90%) than in the muscle. Therefore, we conducted an additional general linear mixed model (GLMM) analysis where we used MeHg-corrected values for liver data. For the analysis, we used measured values for MeHg concentrations for a subset of ringed seal liver (*n* = 13). We calculated MeHg concentrations in the liver of Arctic cod, sculpin, and common eider by multiplying the THg concentration in the liver by the average proportion of MeHg in the liver for each species (0.75, 0.50, and 0.81, respectively) reported in literature (Harley et al. 2015; Wayland et al. 2001). When we refer to models that include both THg and MeHg data, we simply use “Hg” when describing the results.

Some Cd concentrations in the muscle of fish and ringed seal were below analytical detection limits. For ringed seal muscle data, we followed the 2006 US EPA Guideline and substituted non-detected samples with 1/2 the detection limit because < 15% of the samples were below the analytical detection limit (Shoari and Dubé 2018; USEPA 2006). Over 50% of the Arctic cod and sculpin muscle samples were below the analytical detection limits (*n* = 14 of 16 and *n* = 11 of 15, respectively), and standard data estimation/adjustment techniques could not be performed on the left-censored data (Shoari and Dubé 2018; USEPA 2006); therefore, we removed the muscle Cd data of Arctic cod and sculpin from analyses but did use liver Cd data for these species in relevant analyses.

We conducted correlation and regression analyses, respectively, to examine co-variance of metal concentrations between tissues of the same individual and to examine the influence of age or size on metal concentrations (Table S5). We conducted GLMMs to investigate food web patterns of metal concentrations. We included dietary tracers, specifically, δ^15^N, δ^13^C, and δ^34^S ratios as fixed effects, and we included location of sample collection as a random effect. We conducted two GLMMs (one for Cd and one for Hg) using muscle concentrations for vertebrates, whole-body concentrations for blue mussel, and gonad concentrations for sea urchin. We then conducted two more GLMMs using lipid-normalized concentrations in the liver for vertebrates, whole-body concentrations for blue mussel, and gonad concentrations for sea urchin.

Finally, we calculated the trophic magnification slopes (TMS) for both metals in the EHB food web. We ran simple linear regressions of log metal concentrations versus δ^15^N ratios. We used metal concentrations in the muscle for vertebrate species. Additional analyses completed using zooplankton and prey item samples are presented in Appendix A—Supplementary Information.

## Results

### Metal concentrations in marine biota

Metal concentrations varied widely among biota and between tissue types from the same species (Table 1). Average Cd concentrations in biota ranged more than three orders of magnitude from 0.006 to 24.0 µg/g dw. Average Cd concentrations were highest in ringed seal and in common eider liver and were lowest in fish muscle. Average THg concentrations in biota ranged from 0.067 to 20.3 µg/g dw, with the highest levels observed in ringed seal and common eider liver and the lowest levels in sea urchin gonads. Interestingly, both metals were more concentrated in the liver than in the muscle of vertebrate animals, with the exception of THg in fish. For both fish species, THg concentrations were higher in the muscle than in the liver.

**Table 1:** Average Cd, THg, and MeHg concentrations in tissues by species and sampling location. Metal concentrations are presented as average dry weight concentrations ± one standard deviation (SD). Hyphen (-) indicates no value available. Sample sizes are provided in parentheses

Species	Location	Tissue	Cd ± SD (*n*) (µg/g DW)	THg ± SD (*n*) (µg/g DW)	MeHg ± SD (*n*) (µg/g DW)
Arctic cod	Inukjuak	Liver	1.70 ± 2.24 (27)	0.087 ± 0.084 (27)	0.073 ± 0.064 (11)
	Inukjuak	Muscle	0.009 ± 0.006 (6)^a^	0.336 ± 0.300 (27)	-
	Kuujjuaraapik	Liver	0.258 ± 0.121 (15)	0.032 ± 0.022 (15)	-
	Kuujjuaraapik	Muscle	0.006 ± 0.000 (5)^a^	0.360 ± 0.224 (15)	-
	Sanikiluaq	Liver	0.301 ± 0.202 (15)	0.042 ± 0.038 (15)	-
	Sanikiluaq	Muscle	0.006 ± 0.000 (5)^a^	0.355 ± 0.302 (15)	-
Blue mussel	Inukjuak	Whole body	5.64 ± 2.77 (16)	0.200 ± 0.066 (16)	0.035 ± 0.010 (11)
	Kuujjuaraapik	Whole body	2.98 ± 0.756 (10)	0.170 ± 0.033 (10)	0.048 ± 0.005 (5)
	Sanikiluaq	Whole body	6.67 ± 2.86 (20)	0.138 ± 0.047 (20)	0.030 ± 0.015 (15)
	Umiujaq	Whole body	6.20 ± 2.53 (6)	0.159 ± 0.033 (6)	0.031 ± 0.009 (6)
Common eider	Inukjuak	Liver	16.5 ± 11.9 (4)	1.17 ± 0.278 (4)	-
	Inukjuak	Muscle	0.849 ± 0.714 (4)	0.258 ± 0.075 (4)	-
	Kuujjuaraapik	Liver	7.01 ± 8.55 (24)	1.23 ± 1.26 (24)	-
	Kuujjuaraapik	Muscle	0.433 ± 1.029 (11)	0.292 ± 0.199 (24)	-
	Sanikiluaq	Liver	17.3 ± 10.2 (16)	1.14 ± 0.572 (16)	-
	Sanikiluaq	Muscle	0.463 ± 0.343 (6)	0.238 ± 0.148 (16)	-
	Umiujaq	Liver	18.0 ± 29.3 (16)	1.08 ± 0.710 (16)	-
	Umiujaq	Muscle	0.774 ± 0.671 (6)	0.275 ± 0.214 (16)	-
Ringed seal	Kuujjuaraapik	Liver	18.7 ± 13.1 (23)	20.3 ± 22.0 (23)	-
	Kuujjuaraapik	Muscle	0.191 ± 0.217 (22)	0.705 ± 0.354 (23)	-
	Sanikiluaq	Liver	24.0 ± 18.6 (16)	17.8 ± 36.7 (16)	1.13 ± 1.56 (14)
	Sanikiluaq	Muscle	0.156 ± 0.100 (7)	0.645 ± 0.778 (7)	-
Sculpin	Inukjuak	Liver	1.38 ± 1.37 (11)	0.142 ± 0.071 (11)	-
	Inukjuak	Muscle	0.007 ± 0.002 (5)^a^	0.557 ± 0.311 (11)	-
	Kuujjuaraapik	Liver	4.27 ± 3.60 (10)	0.182 ± 0.075 (10)	-
	Kuujjuaraapik	Muscle	0.015 ± 0.009 (5)^a^	0.514 ± 0.211 (10)	-
	Sanikiluaq	Liver	1.21 ± 1.55 (10)	0.183 ± 0.142 (10)	-
	Sanikiluaq	Muscle	0.006 ± 0.000 (5)^a^	0.486 ± 0.398 (10)	-
Sea urchin	Inukjuak	Gonad	1.26 ± 0.204 (4)	0.085 ± 0.010 (4)	0.013 ± 0.004 (4)
	Kuujjuaraapik	Gonad	1.06 ± 0.353 (5)	0.069 ± 0.022 (5)	0.014 ± 0.005 (5)
	Sanikiluaq	Gonad	1.21 ± 0.544 (10)	0.067 ± 0.027 (10)	0.012 ± 0.012 (10)

^a^Averages calculated with non-detects = ½ the detection limit

### Biological and geographic drivers of metal concentrations within species

We observed geographic variation in metal concentrations for common eider and blue mussel, which were the only biota collected at all four communities (Table 1). We observed larger location differences for Cd than for THg. Muscle Cd concentrations of common eiders from Kuujjuaraapik (0.433 ± 1.03 µg/g dw) and from Sanikiluaq (0.463 ± 0.343 µg/g dw) were almost half the average concentrations found at Inukjuak (0.849 ± 0.714 µg/g dw) and at Umiujaq (0.774 ± 0.671 µg/g dw). Common eider liver from Kuujjuaraapik had a lower average Cd concentration (7.01 ± 8.55 µg/g dw) than the other three sampling locations. Blue mussels from Kuujjuaraapik also had a lower average Cd concentration (2.98 ± 0.756 µg/g dw) than the other three sampling locations. In contrast, THg concentrations in the muscle and in the liver of common eider were comparable among locations, and we observed minor variation in THg of blue mussel.

Biological variables explained some within-species variation of metal concentrations, though the influences were not consistent among biota or metals. We were unable to conduct these analyses for Cd concentrations in fish muscle because a high number (> 50%) of samples were below the level of detection. Metal concentrations in the muscle were positively correlated with those in the liver from the same animals for both common eider and ringed seal (Table S6). Age was not an important explanatory variable of metal concentrations in fish liver, and a significant positive effect was only observed for Cd in Arctic cod liver (Table 2). Similarly, length was generally not a strong explanatory variable, and positive effects were only found for THg in the liver and muscle of Arctic cod and in the muscle of ringed seal. In contrast, length was negatively correlated with Cd in Arctic cod liver though the association was weak. Axial girth was not an important variable for explaining variation of Cd concentrations in ringed seal liver but was positively related to liver THg concentration. Overall, biological variables more strongly explained THg concentrations in fish and ringed seal (*R*^2^ from 0.15 to 0.76) than Cd concentrations in biota (*R*^2^ ≤ 0.11).

**Table 2:** Results from within-species multiple regression models of log-transformed Cd and Hg concentrations (µg/g dw) in the liver and muscle in relation to measures of size and age. Significant results are shown in bold. Liver concentrations were lipid normalized

Species/metal/tissue		Estimate (β coef)	SE	*t*-value	Partial *R*^2^	*P*-value
A) Arctic cod—cadmium concentrations (µg/g dw) in the liver						
*R*^2^ = 0.11, *n* = 57, *F*-statistic = 3.348, *P*-value = 0.043						
	(Intercept)	0.224	0.180	1.244	-	0.219
	Age	0.109	0.042	2.571	0.04	**0.013**
	Length	− 0.016	0.008	− 2.102	0.07	**0.040**
B) Arctic cod—mercury concentrations (µg/g dw) in the liver						
*R*^2^ = 0.53, *n* = 57, *F*-statistic = 30.55, *P*-value < 0.001						
	(Intercept)	− 1.750	0.117	− 15.024	-	** < 0.001**
	Age	0.049	0.028	1.800	0.03	0.077
	Length	0.019	0.005	3.769	0.50	** < 0.001**
C) Arctic cod—mercury concentrations (µg/g dw) in the muscle						
*R*^2^ = 0.76, *n* = 57, *F*-statistic = 85.07, *P*-value < 0.001						
	(Intercept)	− 1.709	0.093	− 18.46	-	** < 0.001**
	Age	0.063	0.022	2.882	0.04	**0.006**
	Length	0.025	0.004	6.399	0.72	** < 0.001**
D) Sculpin—cadmium concentrations (µg/g dw) in the liver						
*R*^2^ = 0.03, *n* = 30, *F*-statistic = 0.3924, *P*-value = 0.679						
	(Intercept)	− 0.260	0.654	− 0.397	-	0.694
	Age	− 0.001	0.060	− 0.014	-	0.989
	Length	0.021	0.031	0.690	0.03	0.496
E) Sculpin—mercury concentrations (µg/g dw) in the liver						
*R*^2^ = 0.15, *n* = 30, *F*-statistic = 2.423, *P*-value = 0.108						
	(Intercept)	− 1.466	0.371	− 3.947	-	**0.001**
	Age	− 0.013	0.034	− 0.382	-	0.706
	Length	0.034	0.018	1.911	0.15	0.067
F) Sculpin—mercury concentrations (µg/g dw) in the muscle						
*R*^2^ = 0.26, *n* = 30, *F*-statistic = 4.67, *P*-value = 0.020						
	(Intercept)	− 1.362	0.402	− 3.384	-	**0.002**
	Age	0.033	0.037	0.877	0.02	0.388
	Length	0.032	0.019	1.689	0.24	0.103
G) Ringed seal—cadmium concentrations (µg/g dw) in the liver						
*R*^2^ = 0.06, *n* = 38, *F*-statistic = 1.157, *P*-value = 0.326						
	(Intercept)	0.919	0.353	2.242	-	**0.031**
	Length	0.007	0.006	1.224	0.05	0.220
	Axial Girth	− 0.005	0.007	-0.639	0.01	0.421
H) Ringed seal—mercury concentrations (µg/g dw) in the liver						
*R*^2^ = 0.70, *n* = 38, *F*-statistic = 40.88, *P*-value < 0.001						
	(Intercept)	− 1.268	0.269	− 4.718	-	** < 0.001**
	Length	0.006	0.005	1.296	0.01	0.203
	Axial Girth	0.018	0.006	3.169	0.69	**0.003**
I) Ringed seal—cadmium concentrations (µg/g dw) in the muscle^a, b^						
*R*^2^ = 0.04, *n* = 29, *F*-statistic = 1.044, *P*-value = 0.316						
	(Intercept)	0.005	0.178	0.025	-	0.980
	Length	0.001	0.001	1.022	-	0.316
J) Ringed seal—mercury concentrations (µg/g dw) in the muscle^b^						
*R*^2^ = 0.56, *n* = 30, *F*-statistic = 35.01, *P*-value < 0.001						
	(Intercept)	− 1.252	0.173	− 7.241	-	** < 0.001**
	Length	0.008	0.001	5.917	-	** < 0.001**

^a^Cadmium concentrations were not log-transformed—model assumptions met without data transformation

^b^Axial girth was removed from analysis in the ringed seal muscle regressions due to a VIF value over 5

### Trophic influences on metal concentrations

**Fig. 2:**
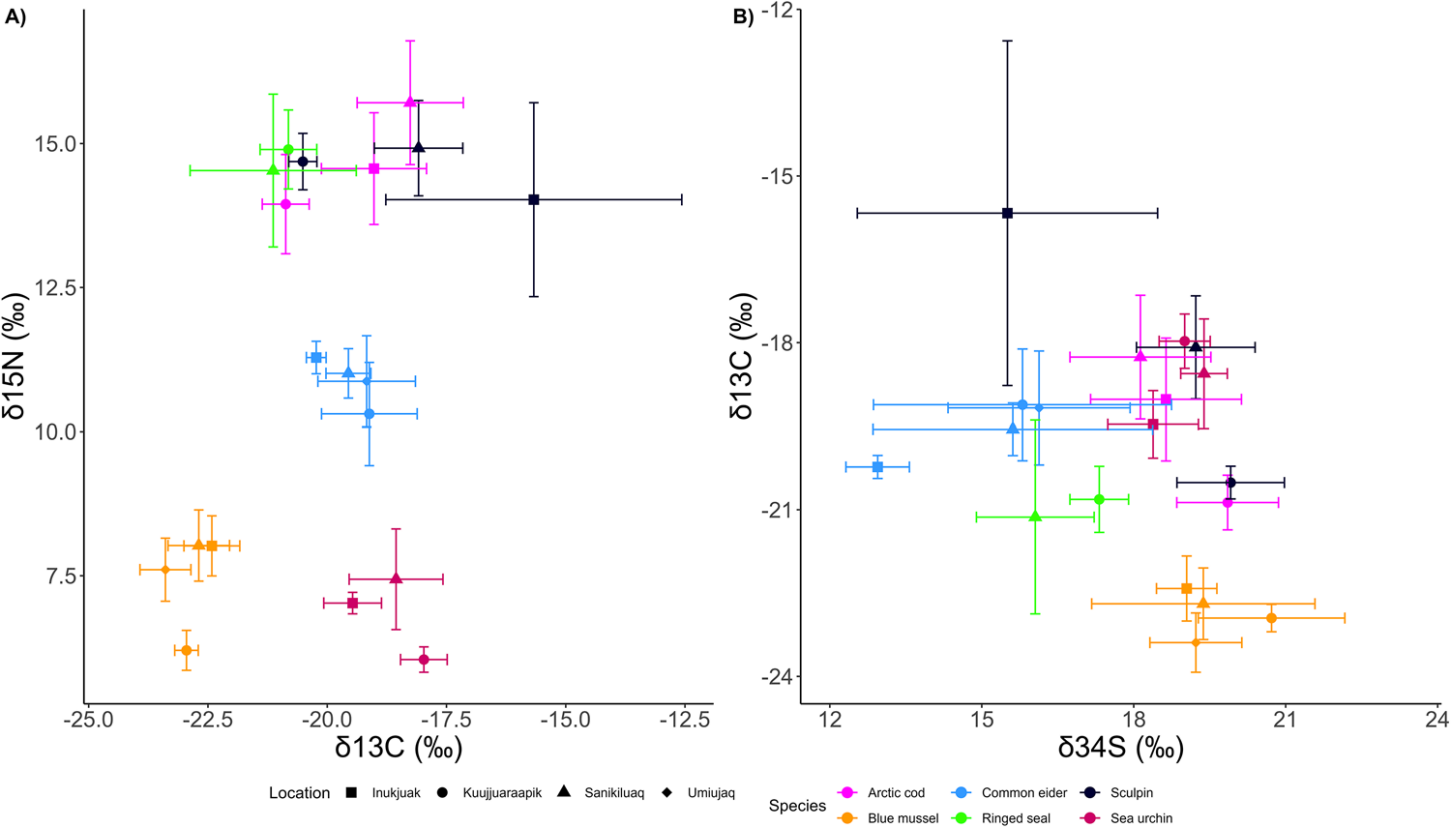
**A**) δ^15^N and δ^13^C and **B**) δ^13^C and δ^34^S stable isotope values of marine biota collected from four locations in east Hudson Bay, Canada. Values are presented as means ± SD

**Fig. 3:**
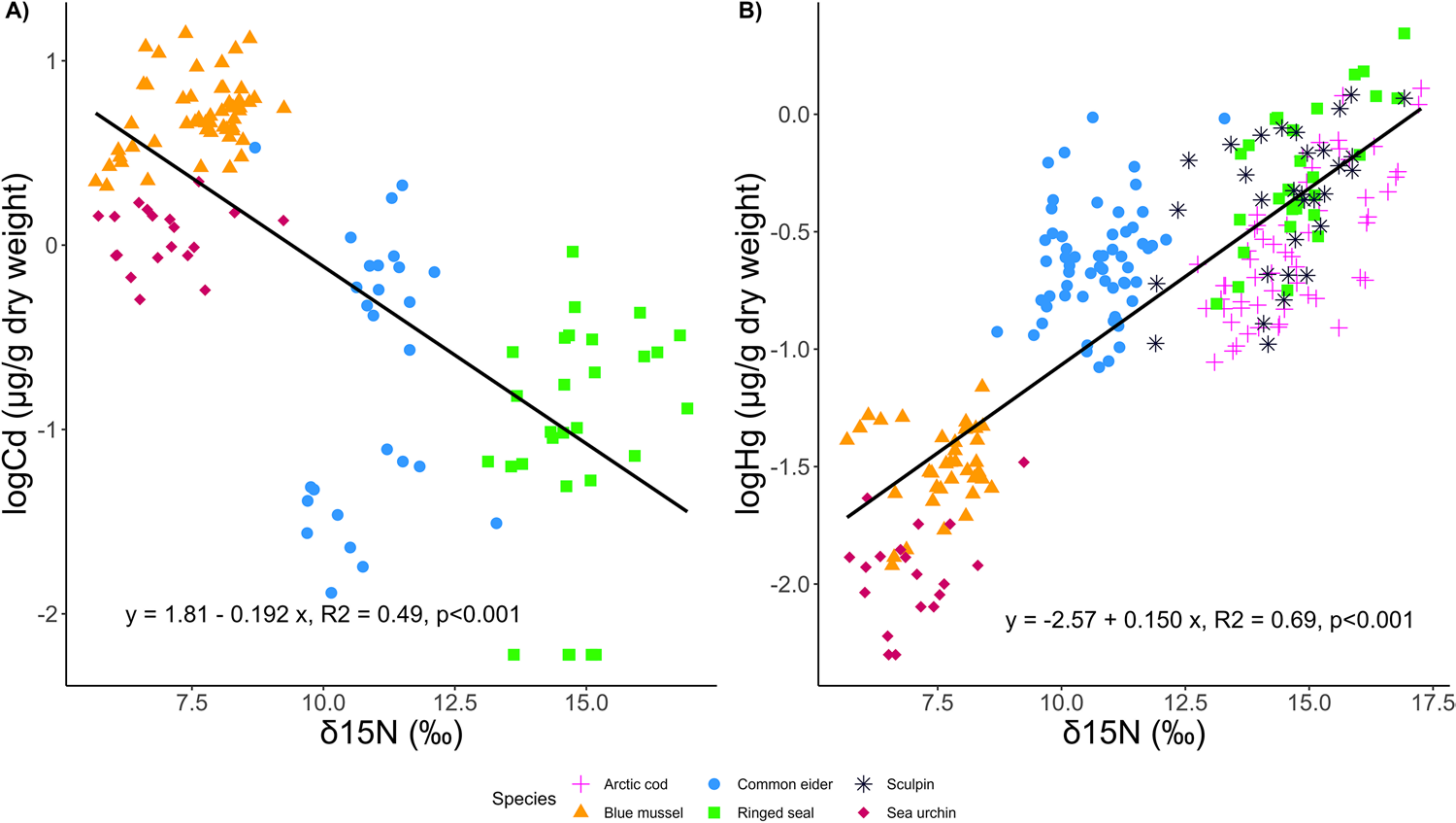
Scatterplots with regression lines of **A**) log Cd concentration and **B**) log Hg concentration in tissues of sample species from east Hudson Bay, Canada, in relation to δ^15^N values. For vertebrate animals, muscle concentrations are plotted

Mean δ^15^N values ranged from 6.0 ± 0.2‰ to 15.7 ± 1.1‰ (Fig. 2, Table S7). Blue mussel and sea urchin had the lowest mean δ^15^N values of sampled species. Arctic cod, ringed seal, and sculpin shared similar trophic positions in this food web as indicated by their similar mean δ^15^N values. Mean δ^13^C values ranged from − 23.4 ± 0.5‰ to − 15.7 ± 3.1‰ in sampled biota (Fig. 2, Table S7). The observed δ^13^C values indicate variation in habitat-specific feeding among sampled species. Mean δ^34^S values ranged from 13.0 ± 0.6‰ to 20.7 ± 1.5‰ (Fig. 2, Table S7). We observed variation in δ^34^S values among species, which indicates differences in foraging behavior. We observed contrasting food web patterns between Cd and Hg when using the muscle for vertebrate species in the GLMM models (Table 3). Cadmium concentrations of biota were negatively correlated with δ^15^N values (an indicator of trophic position), while an expected positive correlation was found for Hg concentrations (Table 3, Fig. 3). These results indicated food web biodilution of Cd in contrast with biomagnification of Hg in the same food web. The TMS for Cd in the EHB food web was − 0.192 ± 0.018 (Table 4, Fig. 3). The TMS for Hg in the EHB food web was 0.150 ± 0.007 (Table 4, Fig. 3). While we did not include Arctic cod and sculpin samples in the analyses using Cd concentrations in vertebrate muscle, Cd concentrations in fish muscle tissue are lower than concentrations of Cd in invertebrates (Table 1), which also indicates biodilution of Cd, even if we were unable to quantify the exact rate of biodilution. Habitat-specific feeding, indicated by a correlation with δ^13^C values, also explained Cd but not Hg concentrations in the food web (Table 3). A negative correlation suggested a greater reliance on pelagic carbon was associated with higher Cd concentrations. Sulfur isotope values explained Hg but not Cd concentrations in the food web. Mercury concentrations were negatively correlated with δ^34^S values.

**Table 3:** Summary of among-species GLMMs explaining differences in log-transformed Cd and Hg concentrations (µg/g dw) in tissues of 6 species from east Hudson Bay. The muscle was used for vertebrate species. Fish data were not included in the Cd GLMM due to > 50% of Cd samples being below the detection limit. The explanatory variables included in the models were δ^15^N, δ^13^C, and δ^34^S. Location of sample collection was included as a random effect. Significant results are shown in bold

Model		Estimate (β coef)	SE	df	*t*-value	*P*-value	Variance	SD
A) logCd ~ δ^15^N + δ^13^C + δ^34^S + (1 | location), *n* = 127								
Fixed effects	(Intercept)	− 2.638	0.816	121.534	− 3.231	**0.002**		
	δ^15^N	− 0.135	0.019	122.993	− 7.143	** < 0.001**		
	δ^13^C	− 0.164	0.027	121.657	− 6.065	** < 0.001**		
	δ^34^S	0.028	0.033	122.931	0.847	0.399		
Random effects	Location						0.084	0.290
	Residual						0.238	0.488
B) logHg ~ δ^15^N + δ^13^C + δ^34^S + (1 | location), *n* = 234								
Fixed effects	(Intercept)	− 1.674	0.232	116.783	− 7.224	** < 0.001**		
	δ^15^N	0.150	0.006	228.230	26.229	** < 0.001**		
	δ^13^C	− 0.007	0.010	228.190	− 0.683	0.495		
	δ^34^S	− 0.057	0.008	227.914	− 7.410	** < 0.001**		
Random effects	Location						0.025	0.159
	Residual						0.066	0.257

**Table 4:** Summary of the trophic magnification slope (TMS) analysis for metal biomagnification in an east Hudson Bay arctic marine food web. Metal concentrations were log-transformed to meet the assumption of normality. The muscle was used for vertebrate species. Arctic cod and sculpin muscle data was not included in the Cd TMS analysis due to > 50% of Cd samples being below the detection limit. Significant results are in bold

Model		Estimate (β coef)	SE	*t*-value	*P*-value
A) logCd ~ δ^15^N					
*R*^2^ = 0.49, *n* = 127, *F*-statistic = 117.9, *P*-value < 0.001					
	(Intercept)	1.805	0.184	9.841	** < 0.001**
	δ^15^N	− 0.192	0.018	− 10.857	** < 0.001**
B) logHg ~ δ^15^N					
*R*^2^ = 0.69, *n* = 234, *F*-statistic = 508.8, *P*-value < 0.001					
	(Intercept)	− 2.568	0.082	− 31.27	** < 0.001**
	δ^15^N	0.150	0.007	22.56	** < 0.001**

**Fig. 4:**
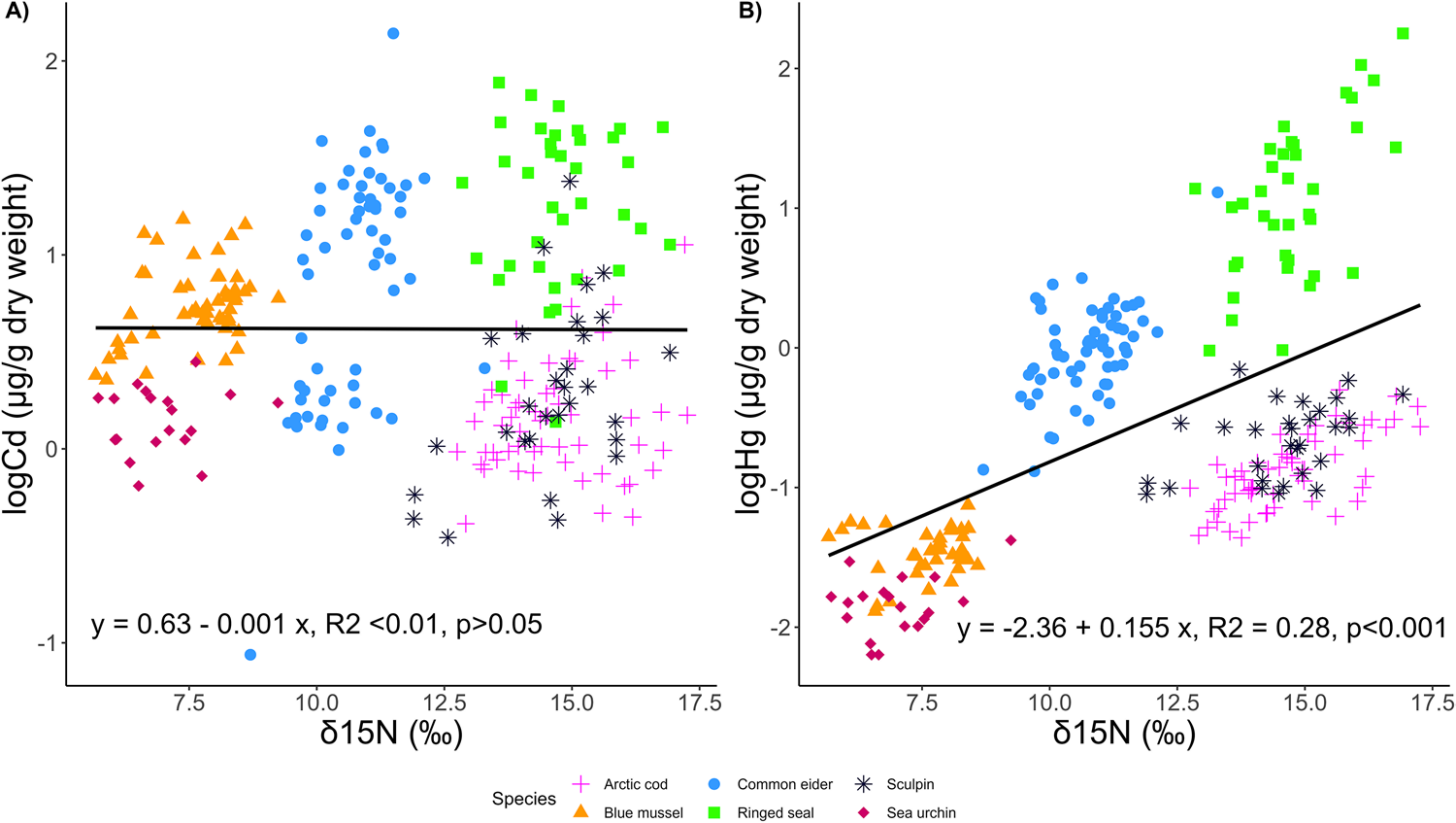
Scatterplots with regression lines of **A**) log Cd concentration and **B**) log Hg concentration in tissues of sample species from east Hudson Bay, Canada, in relation to δ^15^N values. For vertebrate animals, liver concentrations are plotted

We found a different food web pattern for Cd when the GLMM models were run using the liver for vertebrate species (Table 5). Note that this Cd GLMM model included fish liver data (which, unlike fish muscle, had Cd concentrations consistently above analytical detection limits). We observed both Hg and Cd concentrations were positively correlated with δ^15^N (Table 5, Fig. 4). However, the correlation between log Cd concentrations and δ^15^N was weak (ß coef = 0.027 ± 0.010, *P* = 0.006), compared to the correlation between log Hg concentrations and δ^15^N (ß coef = 0.171 ± 0.013, *P* < 0.001), and the former correlation was only significant after accounting for other factors in the model. Liver Cd concentrations ranged more than two orders of magnitude among vertebrate species with a similar trophic position, and there was no simple correlation between liver Cd concentration and δ^15^N (Fig. 4). Additionally, δ^13^C and δ^34^S values were significant explanatory variables for Cd and for Hg concentrations, indicating influences of habitat-specific feeding on metal concentrations in biota (Table 5). Similar to the other models using the muscle (Table 3), liver Cd and liver Hg concentrations were negatively correlated with δ^13^C values, reflecting enhanced bioaccumulation associated with feeding on pelagic carbon sources. Cadmium and Hg concentrations of biota were negatively correlated with δ^34^S values (Table 5), indicating an effect of habitat-specific feeding on metal exposure. The GLMM we ran using MeHg-corrected values for liver data showed the same patterns as the GLMM run using THg concentrations in the liver (Table S8).

**Table 5:** Summary of among-species GLMMs explaining differences in log-transformed Cd and Hg concentrations (µg/g dw) in tissues of 6 species from east Hudson Bay. Liver was used for vertebrate species. Tissue concentrations were lipid normalized. The explanatory variables included in the models were δ^15^N, δ^13^C, and δ^34^S. Location of sample collection was included as a random effect. Significant results are in bold

Model		Estimate (β coef)	SE	df	*t*-value	*P*-value	Variance	SD
A) logCd ~ δ^15^N + δ^13^C + δ^34^S + (1 | location), *n* = 255								
Fixed effects	(Intercept)	− 1.125	0.387	243.797	− 2.910	**0.004**		
	δ^15^N	0.027	0.010	250.985	2.746	**0.006**		
	δ^13^C	− 0.150	0.017	250.994	− 9.038	** < 0.001**		
	δ^34^S	− 0.089	0.013	250.660	− 6.601	** < 0.001**		
Random effects	Location						0.008	0.087
	Residual						0.224	0.474
B) logHg ~ δ^15^N + δ^13^C + δ^34^S + (1 | location), *n* = 240								
Fixed effects	(Intercept)	− 1.511	0.507	125.951	− 2.980	**0.003**		
	δ^15^N	0.171	0.013	234.460	13.668	** < 0.001**		
	δ^13^C	− 0.117	0.021	234.119	− 5.539	** < 0.001**		
	δ^34^S	− 0.189	0.017	234.857	− 11.398	** < 0.001**		
Random effects	Location						0.113	0.335
	Residual						0.336	0.579

## Discussion

Trophic transfer patterns for Cd depended on the vertebrate tissue examined. We observed food web biodilution for muscle Cd concentrations of marine vertebrates. This finding was consistent with Cd biodilution reported in other aquatic food webs (Campbell et al. 2005; Cardwell et al. 2013; Pantoja-Echevarría et al. 2023; Sun et al. 2020) and contrasts with Cd biomagnification reported for Arctic seabirds (Øverjordet et al. 2015) and for a benthic coastal food web (Croteau et al. 2005). Liver Cd concentrations of marine vertebrates showed a weak positive correlation with trophic position, though only for some species. In contrast, we observed strong Hg biomagnification in the food web for both the muscle and liver. The contrasting trends observed for Cd and Hg transfer in the EHB food web suggest that biomagnification is not a likely explanation for elevated Cd concentrations in the liver of some Arctic marine vertebrates (e.g., Dietz et al. 1998; Mallory et al. 2004, 2014, 2017). Rather, our findings suggest that tissue- and species-specific physiology may more accurately explain Cd concentration patterns. Our study indicates that tissue type and the inclusion of lower trophic level biota are important for investigating food web processes, and caution is warranted when interpreting Cd biomagnification in marine biota over a narrow range in trophic position (Chevrollier et al. 2022; Øverjordet et al. 2015; Tian et al. 2022).

Tissue- and species-specific physiology can explain the patterns of Cd concentrations in this Arctic marine food web. The discrepancy in trends observed between the liver and muscle is likely due to Cd detoxification in the vertebrate study species (Gao et al. 2021). The muscle consistently has lower Cd concentrations than the liver and kidney in fish and in wildlife (Dietz et al. 1996; Wayland and Scheuhammer 2011). This difference may be due to higher Cd depuration rates in the muscle relative to the liver, at least for fishes (de Conto Cinier et al. 1999; Pavlaki et al. 2021; Soegianto et al. 2022). In vertebrate animals, Cd is detoxified through binding to metallothionein proteins and storage in the liver and in the kidney (Wayland and Scheuhammer 2011). Subcellular measurements of Cd indicate that this metal is largely bound as stable metallothionein complexes in the liver (Desjardins et al. 2022; Monteiro et al. 2019). These findings explain why we observed such low Cd concentrations in fish muscle but detected Cd in fish liver and why we found the highest Cd concentrations in the liver of ringed seal and common eider. Elevated liver Cd concentrations likely reflect greater detoxification and storage of this metal, instead of greater dietary exposure to top predators due to biomagnification. The differences in Cd concentrations that we observed between Arctic marine vertebrates may also be due to species-specific physiology, such as differences in metallothionein production and the rate of detoxification in the liver (Le Croizier et al. 2019; Lucia et al. 2012). In contrast, we found higher concentrations of Hg in both the liver and muscle of vertebrate species, which in this case is attributed to biomagnification because of the strong positive correlations with trophic position.

We observed variation in feeding habitats: benthic versus pelagic, indicated by variation in δ^13^C values, and sulfate-reducing versus oxidizing environments, indicated by variation in δ^34^S values. Foraging behavior influenced metal concentrations in sampled biota. Pelagic feeding was associated with higher Cd concentrations in the Hudson Bay food web. This finding is consistent with studies of another Arctic marine food web (Campbell et al. 2005) and of pinnipeds on the Uruguayan coast (De María et al. 2021). Polar crustaceans can have elevated Cd concentrations and may be an important dietary source of Cd to marine vertebrates (Lischka et al. 2020; Macdonald et al. 2000; Macdonald and Sprague 1988). However, our findings differ from a study that measured Cd in ringed seal across the Canadian Arctic, where liver concentrations increased with relative carbon source, indicating benthic feeding enhanced Cd bioaccumulation (Brown et al. 2016). Nevertheless, the same study found that seals which fed primarily on pelagic amphipods near one of the communities in our study area (Inukjuak) had the highest measured Cd concentrations (Brown et al. 2016). Thus, it could be the case that some fish and some ringed seal in our study were feeding more on pelagic invertebrates leading to overall higher concentrations of Cd in their tissues. We similarly observed that pelagic feeding was associated with greater Hg concentrations in vertebrate species, which has also been reported for other Arctic marine food webs (Campbell et al. 2005; Hilgendag et al. 2022). In the Arctic Ocean, Hg methylation occurs in the water column and the highest seawater methylmercury concentrations occur at subsurface depths where planktonic uptake may be important (Jonsson et al. 2022; Wang et al. 2018).

We found a negative relationship between metal concentrations and δ^34^S values, which indicates an effect of foraging habitat on exposure. The pattern we observed is consistent with another Hg study conducted in northern Hudson Bay on Arctic seabird prey (Góngora et al. 2018). However, positive and non-significant correlations between metal concentrations and δ^34^S values in marine biota have also been reported (García Barcia et al. 2021; Lippold et al. 2020), which may be due to the influence of multiple processes on environmental δ^34^S gradients. The δ^34^S values of marine wildlife can be influenced by foraging in estuarine to saline transitions, benthic or pelagic habitats, and sulfate-reducing environments (Chételat et al. 2020). In our study, biota were collected from an entirely marine environment; therefore, we make the assumption that variation in δ^34^S values is more likely due to differences in degrees of sulfate reduction across foraging habitats rather than due to terrestrial or estuarine influences. Amiraux et al. (2023) observed high variability in δ^34^S values among benthic invertebrates sampled from Hudson Bay and noted lower δ^34^S values in species that fed within the sediment layer where sulfate reduction occurs. A similar trend was observed in species sampled from the Baltic Sea, where δ^34^S values helped further distinguish between epibenthic and infaunal food sources of the benthic-feeding velvet scoter (*Melanitta fusca*) (Morkūnė et al. 2018). We observed higher δ^34^S values in blue mussel and sea urchin which are both epibenthic species that feed above the sediment layer and lower δ^34^S values in eider and ringed seal which feed to some extent on infaunal taxa. Ringed seal may also feed on benthic fish such as sculpin that feed on infaunal taxa. Therefore, the negative relationship between metal concentrations and δ^34^S values we observed may suggest that feeding on fauna inhabiting sulfate-reducing environments may enhance metal exposure. Sulfate reduction is associated with greater MeHg production (Gilmour et al. 1992); therefore, biota feeding in areas where sulfate reduction occurs may be exposed to higher levels of MeHg. However, the relationship between foraging in habitats with higher degrees of sulfate reduction and increased cadmium bioaccumulation is unclear and warrants further investigation. Overall, sulfur isotope ratios provide complementary information to carbon isotope ratios because they help further distinguish benthic feeding by tracing consumption of infaunal versus epibenthic food sources.

We observed location effects in this study, specifically common eider livers and blue mussels sampled at Kuujjuaraapik had approximately half the average Cd concentrations of those biota sampled at the other three sites. The lower Cd concentrations observed in eider and in blue mussel suggest that there may be spatial differences in the bioavailability of Cd in east Hudson Bay. Uptake and depuration rates of Cd in marine invertebrates can be influenced by water quality variables including salinity, temperature, and pH (Pavlaki et al. 2017; Rainbow and Luoma 2011). Therefore, possible differences in water chemistry between sites may be contributing to the observed differences in blue mussel Cd concentrations, though further research is needed to evaluate the environmental drivers of spatial patterns in metal bioaccumulation.

Trophic magnification slopes have been widely measured to evaluate Hg biomagnification in food webs (Lavoie et al. 2013). Biomagnification rates of Hg, based on TMS values, can differ by latitude, environment (e.g., marine and fresh water), and even between habitats connected to the same food web (Hilgendag et al. 2022; Lavoie et al. 2013). The Hg TMS measured in this study (0.15 ± 0.01) is within the range of other studies of Arctic marine food webs (0.10–0.25) that included seabirds and marine mammals (as summarized in Hilgendag et al. (2022)). Few TMS values have been reported for Cd in Arctic marine food webs, and the value of − 0.19 ± 0.02 measured in this study is comparable, though steeper than the TMS (− 0.09) of Campbell et al. (2005). Signa et al. (2017, 2019) reported Cd TMS values of − 0.08 and − 0.07 for a Mediterranean food web and an Antarctic food web, respectively. The ecotoxicological significance of this variation in Cd biodilution rate is unclear.

Dietary exposure to Cd can be harmful to both humans and wildlife leading to kidney, bone, and/or reproductive damage (ATSDR 2012; Marettová et al. 2015; Thévenod and Lee 2013; Wayland and Scheuhammer 2011). In the Hudson Bay food web, average Cd concentrations in tissues were below adverse effect thresholds for sampled species (birds, 45–70 µg/g ww in the liver (Wayland and Scheuhammer 2011); marine mammals, 200 µg/g ww (AMAP 2005)). Exposure to the organic form of mercury (MeHg) is hazardous to human and to wildlife health. Methylmercury may pass the blood–brain barrier and cause neurological damage via oxidative stress, neuro-inflammation, cell death, neurogenesis impairment, calcium imbalance, DNA damage, changes to glutamate metabolism, and/or changes to neurotransmission (summarized in Novo et al. 2021). In the Hudson Bay marine food web, average Hg concentrations in tissues of sampled species were below wildlife adverse effect thresholds (fish, 0.5 µg/g ww in the muscle (Sandheinrich and Wiener 2011); birds, 20 µg/g ww in the liver (Shore et al. 2011); marine mammals, 61 µg/g ww in the liver (Rawson et al. 1993)). Overall, our data suggest that Arctic marine wildlife were at low risk of experiencing sublethal toxicological effects of Cd and Hg exposure, consistent with earlier assessment (Dietz et al. 1998; Fisk et al. 2005). However, our comparison with tissue burden thresholds provides only a preliminary scoping of toxicological risk, and more subtle subchronic effects, such as suppression of the immune system, may occur at lower tissue concentrations (Desforges et al. 2016).

## Conclusions

This study contributes to broader research on trophic transfer of Cd in marine food webs by presenting findings for the understudied Arctic Ocean. Large variation in Cd and Hg concentrations, which ranged three orders of magnitude in the Arctic marine food web of east Hudson Bay, was related to trophic position and foraging habitat. Mercury biomagnified predictably through the food web, whereas the trophic transfer patterns of Cd differed by vertebrate tissue type. Biodilution of Cd in the food web (based on vertebrate muscle) indicated that elevated Cd in the liver of ringed seal and common eider could not be explained by greater dietary exposure. Rather, elevated Cd concentrations in some Arctic marine mammals and seabirds are likely due to Cd detoxification and storage in the liver. Species-specific physiology may also play a role in controlling Cd tissue burdens, and future research could characterize detoxification processes for Arctic marine vertebrates, focusing on metallothionein production and subcellular partitioning of Cd.

### Supplementary Information

Below is the link to the electronic supplementary material.

Supplementary file1 (DOCX 106 KB)

## Data Availability

The data used in this study are available from the Open Data Portal of the Government of Canada at https://data-donnees.ec.gc.ca/data/substances/monitor/a-east-hudson-bay-network-research-initiative-on-regional-metal-accumulation-in-the-marine-food-web/?lang=en.

## References

[CR1] Agency for Toxic Substances and Disease Registry (ATSDR) (2012). Toxicological profile for Cadmium.

[CR2] AMAP (2005) AMAP Assessment 2002: Heavy metals in the Arctic. Arctic Monitoring and Assessment Programme, Oslo, Norway. xvi + 265 pp. https://www.amap.no/documents/doc/amap-assessment-2002-heavy-metals-in-the-arctic/97

[CR3] AMAP (2021) 2021 AMAP Mercury Assessment: Summary for Policy-makers. Arctic Monitoring and Assessment Programme, Tromsø, Norway. 16 pp. https://www.amap.no/documents/doc/2021-amap-mercury-assessment.-summary-for-policy-makers/3510

[CR4] Amiraux R, Mundy CJ, Pierrejean M (2023). Tracing carbon flow and trophic structure of a coastal Arctic marine food web using highly branched isoprenoids and carbon, nitrogen and sulfur stable isotopes. Ecol Indic.

[CR5] Amlund H, Lundebye A-K, Berntssen MHG (2007). Accumulation and elimination of methylmercury in Atlantic cod (*Gadus morhua L.)* following dietary exposure. Aquat Toxicol.

[CR6] Arai T, Ikemoto T, Hokura A, Terada Y, Kunito T, Tanabe S, Nakai I (2004). Chemical forms of mercury and cadmium accumulated in marine mammals and seabirds as determined by XAFS analysis. Environ Sci Technol.

[CR7] Braune B, Chételat J, Amyot M (2015). Mercury in the marine environment of the Canadian Arctic: review of recent findings. Sci Total Environ.

[CR8] Braune BM, Malone BJ, Burgess NM et al (1999) Chemical residues in waterfowl and gamebirds harvested in Canada, 1987–95. Technical Report Series No. 326. Canadian Wildlife Service, Ottawa, Canada. https://publications.gc.ca/collections/Collection/CW69-5-326E-1.pdf

[CR9] Brown TM, Fisk AT, Wang X, Ferguson SH, Young BG, Reimer KJ, Muir DCG (2016). Mercury and cadmium in ringed seals in the Canadian Arctic: influence of location and diet. Sci Total Environ.

[CR10] Buckley TW, Whitehouse GA (2017). Variation in the diet of Arctic cod (*Boreogadus saida*) in the Pacific Arctic and Bering Sea. Environ Biol Fish.

[CR11] Burger J (2008). Assessment and management of risk to wildlife from cadmium. Sci Total Environ.

[CR12] Campbell LM, Norstrom RJ, Hobson KA, Muir DCG, Backus S, Fisk AT (2005). Mercury and other trace elements in a pelagic Arctic marine food web (Northwater Polynya, Baffin Bay). Sci Total Environ.

[CR13] Cardwell RD, DeForest DK, Brix KV, Adams WJ (2013) Do Cd, Cu, Ni, Pb, and Zn Biomagnify in Aquatic Ecosystems? In: Whitacre DM (ed) Reviews of Environmental Contamination and Toxicology. Springer New York, pp 101–122. 10.1007/978-1-4614-6898-1_4 10.1007/978-1-4614-6898-1_4 23625131

[CR14] Chambellant M, Stirling I, Ferguson S (2013). Temporal variation in western Hudson Bay ringed seal *Phoca hispida* diet in relation to environment. Mar Ecol Prog Ser.

[CR15] Chételat J, Ackerman JT, Eagles-Smith CA, Hebert CE (2020). Methylmercury exposure in wildlife: a review of the ecological and physiological processes affecting contaminant concentrations and their interpretation. Sci Total Environ.

[CR16] Cheung MS, Wang WX (2008). Analyzing biomagnification of metals in different marine food webs using nitrogen isotopes. Mar Pollut Bull.

[CR17] Chevrollier LA, Koski M, Søndergaard J, Trapp S, Aheto DW, Darpaah G, Nielsen TG (2022). Bioaccumulation of metals in the planktonic food web in the Gulf of Guinea. Mar Pollut Bull.

[CR18] Croteau MN, Luoma SN (2008). A biodynamic understanding of dietborne metal uptake by a freshwater invertebrate. Environ Sci Technol.

[CR19] Croteau MN, Luoma SN, Stewart AR (2005). Trophic transfer of metals along freshwater food webs: evidence of cadmium biomagnification in nature. Limnol Oceanogr.

[CR20] de Conto Cinier C, Petit-Ramel M, Faure R, Garin D, Bouvet Y (1999). Kinetics of cadmium accumulation and elimination in carp
* Cyprinus carpio
* tissues. Comp Biochem Physiol C Toxicol Pharmaco.

[CR21] De María M, Szteren D, García-Alonso J, de Rezende CE, Araújo Gonçalves R, Godoy JM, Barboza FR (2021). Historic variation of trace elements in pinnipeds with spatially segregated trophic habits reveals differences in exposure to pollution. Sci Total Environ.

[CR22] De Vera J, Chandan P, Landing WM, Stupple GW, Steffen A, Bergquist BA (2021). Amount, sources, and dissolution of aerosol trace elements in the Canadian Arctic. ACS Earth Space Chem.

[CR23] Desforges JPW, Sonne C, Levin M, Siebert U, De Guise S, Dietz R (2016). Immunotoxic effects of environmental pollutants in marine mammals. Environ Int.

[CR24] Desjardins K, Khadra M, Caron A, Ponton DE, Rosabal M, Amyot M (2022). Significance of chemical affinity on metal subcellular distribution in yellow perch (*Perca flavescens*) livers from Lake Saint-Pierre (QUEBEC, Canada). Environ Pollut.

[CR25] Dietz R, Riget F, Johansen P (1996). Lead, cadmium, mercury and selenium in Greenland marine animals. Sci Total Environ.

[CR26] Dietz R, Nørgaard J, Hansen JC (1998). Have arctic marine mammals adapted to high cadmium levels?. Mar Pollut Bull.

[CR27] Eastwood RA, Macdonald RW, Ehn JK, Heath J, Arragutainaq L, Myers PG, Barber DG, Kuzyk ZA (2020). Role of river runoff and sea ice brine rejection in controlling stratification throughout winter in southeast Hudson Bay. Estuaries Coasts.

[CR28] Espejo W, Padilha JA, Kidd KA, Dorneles PR, Barra R, Malm O, Chiang G, Celis JE (2018). Trophic transfer of cadmium in marine food webs from Western Chilean Patagonia and Antarctica. Mar Pollut Bull.

[CR29] Fisk AT, de Wit CA, Wayland M (2005). An assessment of the toxicological significance of anthropogenic contaminants in Canadian arctic wildlife. Sci Total Environ.

[CR30] Fry B (2013). Using stable CNS isotopes to evaluate estuarine fisheries condition and health. Isotopes Environ Health Stud.

[CR31] Galaktionov KV, Węsławski JM, Stempniewicz L (2021). Food chain, parasites and climate changes in the high Arctic: a case study on trophically transmitted parasites of common eider *Somateria mollissima* at Franz Josef Land. Polar Biol.

[CR32] Gao Y, Wang R, Li Y, Ding X, Jiang Y, Feng J, Zhu L (2021). Trophic transfer of heavy metals in the marine food web based on tissue residuals. Sci Total Environ.

[CR33] García Barcia L, Pinzone M, Lepoint G, Pau C, Das K, Kiszka JJ (2021). Factors affecting mercury concentrations in two oceanic cephalopods of commercial interest from the southern Caribbean. Mar Pollut Bull.

[CR34] Gilmour CC, Henry EA, Mitchell R (1992). Sulfate stimulation of mercury methylation in freshwater sediments. Environ Sci Technol.

[CR35] GOC (2022) A East Hudson Bay Network research initiative on regional metal accumulation in the marine food web. Open data record of the Government of Canada (GOC). https://data-donnees.ec.gc.ca/data/substances/monitor/a-east-hudson-bay-network-research-initiative-on-regional-metal-accumulation-in-the-marine-food-web/?lang=en

[CR36] Góngora E, Braune BM, Elliott KH (2018). Nitrogen and sulfur isotopes predict variation in mercury levels in Arctic seabird prey. Mar Pollut Bull.

[CR37] Gray JS (2002). Biomagnification in marine systems: the perspective of an ecologist. Mar Pollut Bull.

[CR38] Gupta K, Mukhopadhyay A, Babb DG, Barber DG, Ehn JK (2022). Landfast sea ice in Hudson Bay and James Bay. Elementa.

[CR39] Habicht KS, Canfield DE (1997). Sulfur isotope fractionation during bacterial sulfate reduction in organic-rich sediments. Geochim Cosmochim Acta.

[CR40] Harley J, Lieske C, Bhojwani S, Castellini JM, López JA, O’Hara TM (2015). Mercury and methylmercury distribution in tissues of sculpins from the Bering Sea. Polar Biol.

[CR41] Hilgendag IR, Swanson HK, Lewis CW, Ehrman AD, Power M (2022). Mercury biomagnification in benthic, pelagic, and benthopelagic food webs in an Arctic marine ecosystem. Sci Total Environ.

[CR42] Houserová P, Kubáň V, Kráčmar S, Sitko J (2007). Total mercury and mercury species in birds and fish in an aquatic ecosystem in the Czech Republic. Environ Pollut.

[CR43] Jonsson S, Nerentorp Mastromonaco MG, Gårdfeldt K, Mason RP (2022). Distribution of total mercury and methylated mercury species in Central Arctic Ocean water and ice. Mar Chem.

[CR44] Lambelet M, Rehkämper M, van de Flierdt T, Xue Z, Kreissig K, Coles B, Porcelli D, Andersson P (2013). Isotopic analysis of Cd in the mixing zone of Siberian rivers with the Arctic Ocean-new constraints on marine Cd cycling and the isotope composition of riverine Cd. Earth Planet Sci Lett.

[CR45] Landry JJ, Fisk AT, Yurkowski DJ, Hussey NE, Dick T, Crawford RE, Kessel ST (2018). Feeding ecology of a common benthic fish, shorthorn sculpin (*Myoxocephalus scorpius*) in the high arctic. Polar Biol.

[CR46] Lavoie RA, Jardine TD, Chumchal MM, Kidd KA, Campbell LM (2013). Biomagnification of mercury in aquatic food webs: a worldwide meta-analysis. Environ Sci Technol.

[CR47] Le Croizier G, Lacroix C, Artigaud S (2019). Metal subcellular partitioning determines excretion pathways and sensitivity to cadmium toxicity in two marine fish species. Chemosphere.

[CR48] Lehnherr I (2014). Methylmercury biogeochemistry: a review with special reference to Arctic aquatic ecosystems. Environ Rev.

[CR49] Lippold A, Aars J, Andersen M (2020). Two decades of mercury concentrations in Barents Sea polar bears (*Ursus maritimus*) in relation to dietary carbon, sulfur, and nitrogen. Environ Sci Technol.

[CR50] Lischka A, Lacoue-Labarthe T, Bustamante P, Piatkowski U, Hoving HJT (2020). Trace element analysis reveals bioaccumulation in the squid *Gonatus fabricii* from polar regions of the Atlantic Ocean. Environ Pollut.

[CR51] López-García I, Vicente-Martínez Y, Hernández-Córdoba M (2014). Determination of cadmium and lead in edible oils by electrothermal atomic absorption spectrometry after reverse dispersive liquid–liquid microextraction. Talanta.

[CR52] Lucia M, Bocher P, Cosson RP, Churlaud C, Bustamante P (2012). Evidence of species-specific detoxification processes for trace elements in shorebirds. Ecotoxicol.

[CR53] Macdonald C, Sprague J (1988). Cadmium in marine invertebrates and arctic cod in the Canadian Arctic. Distribution and ecological implications. Mar Ecol Prog Ser.

[CR54] Macdonald RW, Barrie LA, Bidleman TF (2000). Contaminants in the Canadian Arctic: 5 years of progress in understanding sources, occurrence and pathways. Sci Total Environ.

[CR55] Mallory ML, Wayland M, Braune BM, Drouillard KG (2004). Trace elements in marine birds, arctic hare and ringed seals breeding near Qikiqtarjuaq, Nunavut, Canada. Mar Pollut Bull.

[CR56] Mallory ML, Braune BM, Robertson GJ, Gilchrist HG, Mallory CD, Forbes MR, Wells R (2014). Increasing cadmium and zinc levels in wild common eiders breeding along Canada’s remote northern coastline. Sci Total Environ.

[CR57] Mallory CD, Gilchrist HG, Robertson GJ, Provencher JF, Braune BM, Forbes MR, Mallory ML (2017). Hepatic trace element concentrations of breeding female common eiders across a latitudinal gradient in the eastern Canadian Arctic. Mar Pollut Bull.

[CR58] Marettová E, Maretta M, Legáth J (2015). Toxic effects of cadmium on testis of birds and mammals: a review. Anim Reprod Sci.

[CR59] Monteiro F, Lemos LS, de Moura JF (2019). Subcellular metal distributions and metallothionein associations in rough-toothed dolphins (*Steno bredanensis*) from Southeastern Brazil. Mar Pollut Bull.

[CR60] Morkūnė R, Lesutienė J, Morkūnas J, Barisevičiūtė R (2018). Triple stable isotope analysis to estimate the diet of the velvet scoter (*Melanitta fusca*) in the Baltic Sea. PeerJ.

[CR61] Ng TYT, Wood CM (2008). Trophic transfer and dietary toxicity of Cd from the oligochaete to the rainbow trout. Aquat Toxicol.

[CR62] Novo JP, Martins B, Raposo RS, Pereira FC, Oriá RB, Malva JO, Fontes-Ribeiro C (2021). Cellular and molecular mechanisms mediating methylmercury neurotoxicity and neuroinflammation. IJMS.

[CR63] Øverjordet IB, Gabrielsen GW, Berg T (2015). Effect of diet, location and sampling year on bioaccumulation of mercury, selenium and cadmium in pelagic feeding seabirds in Svalbard. Chemosphere.

[CR64] Pantoja-Echevarría LM, Marmolejo-Rodríguez AJ, Galván-Magaña F (2023). Trophic structure and biomagnification of cadmium, mercury and selenium in brown smooth hound shark (*Mustelus henlei*) within a trophic web. Food Webs.

[CR65] Pavlaki MD, Morgado RG, van Gestel CAM, Calado R, Soares AMVM, Loureiro S (2017). Influence of environmental conditions on the toxicokinetics of cadmium in the marine copepod *Acartia tonsa*. Ecotoxicol Environ Saf.

[CR66] Pavlaki MD, Morgado RG, Ferreira V, Rocha RJM, Soares AMVM, Calado R, Loureiro S (2021). Cadmium accumulation and kinetics in *Solea senegalensis* tissues under dietary and water exposure and the link to human health. Water.

[CR67] Pedro S, Fisk AT, Ferguson SH, Hussey NE, Kessel ST, McKinney MA (2019). Limited effects of changing prey fish communities on food quality for aquatic predators in the eastern Canadian Arctic in terms of essential fatty acids, methylmercury and selenium. Chemosphere.

[CR68] Post DM (2002). Using stable isotopes to estimate trophic position: models, methods, and assumptions. Ecology.

[CR69] R Core Team (2021) R: A language and environment for statistical computing. R Foundation for Statistical Computing, Vienna, Austria. https://www.R-project.org/

[CR70] Rainbow PS, Luoma SN (2011) Trace Metals in Aquatic Invertebrates. In: Beyer WN, Meador JP (eds) Environmental Contaminants in Biota, 2nd Edition. CRC Press, New York, pp 231–254. 10.1201/b10598-7

[CR71] Rawson AJ, Patton GW, Hofmann S, Pietra GG, Johns L (1993). Liver abnormalities associated with chronic mercury accumulation in stranded Atlantic bottlenosed dolphins. Ecotoxicol Environ Saf.

[CR72] Sandheinrich MB, Wiener JG (2011) Methylmercury in Freshwater Fish. In: Beyer WN, Meador JP (eds) Environmental Contaminants in Biota, 2nd Edition. CRC Press, pp 169–192. 10.1201/b10598-5

[CR73] Schantz MM, Koster BJ, Wise SA, Becker PR (1993). Determination of PCBs and chlorinated hydrocarbons in marine mammal tissues. Sci Total Environ.

[CR74] Shoari N, Dubé JS (2018). Toward improved analysis of concentration data: Embracing nondetects: considering nondetects in concentration data analysis. Environ Toxicol Chem.

[CR75] Shore RF, Pereira MG, Walker LA, Thompson DR (2011) Mercury in Nonmarine Birds and Mammals. In: Beyer WN, Meador JP (eds) Environmental Contaminants in Biota, 2nd Edition. CRC Press, pp 609–626. 10.1201/b10598-19

[CR76] Signa G, Mazzola A, Tramati CD, Vizzini S (2017). Diet and habitat use influence Hg and Cd transfer to fish and consequent biomagnification in a highly contaminated area: Augusta Bay (Mediterranean Sea). Environ Pollut.

[CR77] Signa G, Calizza E, Costantini ML, Tramati C, Sporta Caputi S, Mazzola A, Rossi L, Vizzini S (2019). Horizontal and vertical food web structure drives trace element trophic transfer in Terra Nova Bay, Antarctica. Environ Pollut.

[CR78] Soegianto A, Widyanita A, Affandi M, Wirawan T, Mohamed RMSR (2022). Cadmium and zinc accumulation and depuration in tilapia (*Oreochromis niloticus*) tissues following sub-lethal exposure. Bull Environ Contam Toxicol.

[CR79] Sofyan A, Price DJ, Birge WJ (2007). Effects of aqueous, dietary and combined exposures of cadmium to *Ceriodaphnia dubia*. Sci Total Environ.

[CR80] Søndergaard J, Mosbech A (2022). Mining pollution in Greenland - the lesson learned: a review of 50 years of environmental studies and monitoring. Sci Total Environ.

[CR81] Stewart DB, Barber DG, Ferguson SH, Loseto LL, Mallory ML (2010). The ocean-sea ice-atmosphere system of the Hudson Bay complex. A Little Less Arctic: Top Predators in the World’s Largest Northern Inland Sea, Hudson Bay.

[CR82] Sun T, Wu H, Wang X, Ji C, Shan X, Li F (2020). Evaluation on the biomagnification or biodilution of trace metals in global marine food webs by meta-analysis. Environ Pollut.

[CR83] Szpak P, Buckley M (2020). Sulfur isotopes (δ^34^S) in Arctic marine mammals: indicators of benthic vs. pelagic foraging. Mar Ecol Prog Ser.

[CR84] Thévenod F, Lee WK (2013) Toxicology of cadmium and its damage to mammalian organs. In: Sigel A, Sigel H, Sigel RKO (eds) Cadmium: Toxicity Essentiality, Metal Ions in Life Sciences, vol 11. Springer, Dordrecht, pp 415–490. 10.1007/978-94-007-5179-8_14 10.1007/978-94-007-5179-8_14 23430781

[CR85] Tian J, Lu Z, Sanganyado E, Gan Z, Wang Z, Kong Z, Wu J, Liu W (2022). Tissue distribution and trophic magnification of trace elements in typical marine mammals in Bohai and north Yellow Seas. Mar Pollut Bull.

[CR86] USEPA (2006) Data Quality Assessment: Statistical Methods for Practitioners (EPA QA/G-9S). EPA/240/B-06/003. United States Environmental Protection Agency (USEPA), Office of Environmental Information, Washington, DC. https://www.epa.gov/sites/default/files/2015-08/documents/g9s-final.pdf

[CR87] Wagemann R, Trebacz E, Boila G, Lockhart W (1998). Methylmercury and total mercury in tissues of arctic marine mammals. Sci Total Environ.

[CR88] Wang K, Munson KM, Beaupré-Laperrière A, Mucci A, Macdonald RW, Wang F (2018). Subsurface seawater methylmercury maximum explains biotic mercury concentrations in the Canadian Arctic. Sci Rep.

[CR89] Wayland M, Scheuhammer AM, Lydekker R (2011). Cadmium in Birds. Environmental contaminants in biota.

[CR90] Wayland M, Gilchrist HG, Dickson DL, Bollinger T, James C, Carreno RA, Keating J (2001). Trace elements in king eiders and common eiders in the Canadian Arctic. Arch Environ Contam Toxicol.

[CR91] Zauke GP, Schmalenbach I (2006). Heavy metals in zooplankton and decapod crustaceans from the Barents Sea. Sci Total Environ.

