# Supplementary material for: Contrasting trophic transfer patterns of cadmium and mercury in the Arctic marine food web of east Hudson Bay, Canada
Source: Environ Sci Pollut Res Int. 2024 Feb 20;31(13):20586–600. doi: 10.1007/s11356-024-32268-3 (PMC10927903; doi:10.1007/s11356-024-32268-3)
Supplement: Supplementary file 1 — Supplementary file1 (DOCX 106 KB) [file 11356_2024_32268_MOESM1_ESM.docx]

**Supplementary Information**

**Contrasting trophic transfer patterns of cadmium and mercury in the subarctic marine food web of east Hudson Bay, Canada**

Jillian Rohonczy ^a^, John Chételat ^b^*, Stacey A. Robinson ^b^, Lucassie Arragutainaq ^c^, Joel P. Heath ^d^, Christine McClelland ^b^, Raymond Mickpegak ^e^, Mark R. Forbes ^a^

^a^ Department of Biology, Carleton University, Ottawa, ON K1S 5B6, Canada

^b^ Environment and Climate Change Canada, National Wildlife Research Centre, Ottawa, ON K1A 0H3, Canada

^c^ Sanikiluaq Hunters and Trappers Association, Sanikiluaq, Nunavut XAO 0W0, Canada

^d^ Arctic Eider Society, Sanikiluaq, NU X0A 0W0, Canada

^e^ Sakkuq Landholding Corporation, Kuujjuarapik, QC J0M 1G0, Canada

*** Corresponding author:** John Chételat, email: [john.chetelat@ec.gc.ca](mailto:john.chetelat@ec.gc.ca), Tel. +1 343-809-2741

Table of Contents

[**Table S1.** Mean and standard deviation percent recoveries of six certified reference materials for THg measurement. 4](#_Toc152147088)

[**Table S2.** Mean and standard deviation percent recoveries of four certified reference materials for MeHg measurement. 5](#_Toc152147089)

[**Table S3.** Mean and standard deviation percent recoveries of four certified reference materials for Cd measurement. 6](#_Toc152147090)

[**Statistical Analyses** 7](#_Toc152147091)

[**Table S4.** Results from within-species Kendall and Pearson correlations between lipid % and metal concentrations in liver tissue of Arctic cod and sculpin. Significant results in bolded. Metal concentrations were log transformed for sculpin analyses to meet the assumption of normality. 9](#_Toc152147092)

[**Table S5.** Raw data of Arctic cod, sculpin, and ringed seal age (years), length (cm), and axial girth (cm) measurements. 10](#_Toc152147093)

[**Table S6.** Results from within-species Kendall and Pearson correlations between metal concentrations (µg/g dw), in muscle and in liver. Liver concentrations were lipid normalized. Significant results bolded. 15](#_Toc152147094)

[**Table S7.** Average (± SD) of nitrogen, carbon and sulfur stable isotope values of species sampled from east Hudson Bay by location 16](#_Toc152147095)

[**Table S8.** Summary of among-species GLMMs explaining differences in log-transformed MeHg-corrected concentrations (µg/g dw) in tissues of 6 species from east Hudson Bay. Liver concentrations were used for vertebrate species. Liver concentrations were lipid normalized. The explanatory variables included in the model were δ^15^N, δ^13^C, and δ^34^S. Location of sample collection was included as a random effect. Significant results are shown in bold. 17](#_Toc152147096)

[**Table S9.** Results from among-species Kendall’s correlation analyses between log Cd concentrations and stable-isotopes. Muscle was used for vertebrate species. Arctic cod and sculpin samples were not included due to >50% of Cd samples being below the detection limit. Significant results are shown in bold. n = 127 18](#_Toc152147097)

[**Table S10.** Results from among-species Kendall’s correlation analyses between log Hg concentrations and stable-isotopes. Liver was used for vertebrate species. Significant results are shown in bold. n = 240 19](#_Toc152147098)

[**Table S11.** Average methylmercury concentrations in tissue and average stable isotope values by species and location for amphipod, copepod, and gut content samples. Metal concentrations are recorded as dry weight concentrations ± SD. Hyphen (-) indicates no value available. 20](#_Toc152147099)

[**Table S12.** Summary of among-species GLMMs explaining differences in log Hg concentrations (µg/g dw) in tissues of 12 species from east Hudson Bay. Muscle was used for vertebrates. The explanatory variables included in the model were δ15N, δ13C, and δ34S. Location of sample collection was included as a random effect. Significant results are shown in bold. 21](#_Toc152147100)

[**Table S13.** Summary of trophic magnification slope (TMS) analysis for Hg biomagnification in an east Hudson Bay arctic marine food web. Dataset expanded to include gut content data in analysis. Muscle used for vertebrates. Significant results are shown in bold. 22](#_Toc152147101)

[**Fig. S1** Trophic magnification slope (TMS) of log Hg in east Hudson Bay food web. Using muscle for vertebrates. Dataset expanded to include gut content data in analysis. 23](#_Toc152147102)

# **Table S1.** Mean and standard deviation percent recoveries of six certified reference materials for THg measurement.

| **Certified Reference Material** | **n** | **Mean % recovery** | **SD % recovery** |
| --- | --- | --- | --- |
| JRC BCR-463 Tuna Fish | 19 | 97.3 | 2.0 |
| IAEA-436 Tuna Flesh | 20 | 101.6 | 3.6 |
| NIST 1566b Oyster Tissue | 9 | 86.6 | 2.4 |
| NIST 2976 Mussel Tissue | 7 | 98.0 | 2.7 |
| NRC TORT-3 Lobster Hepatopancreas | 40 | 92.9 | 3.7 |
| NRC DORM-4 Fish Protein | 13 | 95.5 | 2.8 |

# **Table S2.** Mean and standard deviation percent recoveries of four certified reference materials for MeHg measurement.

| **Certified Reference Material** | **n** | **Mean % recovery** | **SD % recovery** |
| --- | --- | --- | --- |
| NRC TORT-3 Lobster Hepatopancreas | 3 | 83.8 | 1.1 |
| NRC DORM-4 Fish | 3 | 88.8 | 1.4 |
| IAEA-436 Fish Flesh | 3 | 104.3 | 2.5 |
| NIST 2976 Mussel Tissue | 6 | 89.5 | 2.1 |

# **Table S3.** Mean and standard deviation percent recoveries of four certified reference materials for Cd measurement.

| **Certified Reference Material** | **n** | **Mean % recovery** | **SD % recovery** |
| --- | --- | --- | --- |
| NRC TORT-3 Lobster Hepatopancreas | 3 | 97.0 | 2.2 |
| IAEA-436 Fish Flesh | 6 | 103.3 | 10.6 |
| NRC DORM-4 Fish | 6 | 98.8 | 1.2 |
| NRC DOLT-5 | 3 | 92.3 | 0.3 |

# **Statistical Analyses**

For the correlation analyses the assumption of normality was tested visually Q-Q plots, and analytically with Shapiro-Wilk normality tests on each variable. We applied non-parametric analyses (Kendall’s tau) in cases where the assumption of normality was not met. For the multiple regression analyses, we investigated the assumption of multivariate normality using Q-Q plots. We tested the assumption of homogeneity of residual variance using Scale-Location plots. We computed Variation Inflation Factors (VIFs) for the explanatory variables to test whether multi-collinearity was present; if an explanatory variable had a VIF value greater than 5 it was removed from the analysis (James et al., 2014). We determined partial r^2^ values for the explanatory variables by calculating the increase in R^2^ after adding each explanatory variable into the model one after the other, starting with the most significant variable. For the GLMM analyses, we checked the assumption of normality with a histogram of the residuals. We verified homogeneity by plotting residuals against fitted values. We assessed independence by plotting residuals against each explanatory variable. We verified that the response variable was a reasonably linear function of the fitted values. In two cases, the assumption of homogeneity of variance was violated; 1) the GLMM run to investigate food web patterns of Cd using vertebrate muscle tissue, and 2) the GLMM run to investigate food web patterns of Hg using vertebrate liver tissue. Therefore, to confirm the results of these two GLMMs, we ran non-parametric correlations (Kendall’s tau) between log metal concentrations and each fixed effect included in the GLMM models. The non-parametric correlations suggest that log Cd concentrations are positively correlated to δ^34^S ratios (Table S8), which is not reflected in the GLMM results. The results from the non-parametric correlations between log Hg concentrations and each fixed effect show the same trends as the GLMM results (Table S9).

We ran an additional GLMM model to investigate differences in Hg concentrations between species using an expanded dataset that included gut content samples (small prey fish and plankton), and pelagic zooplankton samples (Table S10); we used muscle Hg concentrations for vertebrate species in this analysis. Results from the analysis are shown in Table S11. We also ran an additional regression analysis to calculate the trophic magnification slope (TMS) for Hg using the expanded dataset. Results from the analysis are shown in Table S12.

**Results**

# **Table S4.** Results from within-species Kendall and Pearson correlations between lipid % and metal concentrations in liver tissue of Arctic cod and sculpin. Significant results in bolded. Metal concentrations were log transformed for sculpin analyses to meet the assumption of normality.

|  | | **Cadmium** | | **Mercury** | |
| --- | --- | --- | --- | --- | --- |
| Species (Common name) | n | r | P-value | r | P-value |
| Arctic cod | 57 | -0.62^a^ | **<0.001** | -0.48^a^ | **<0.001** |
| Sculpin | 30 | -0.27^b^ | 0.148 | -0.22^b^ | 0.238 |

^a^ Correlation estimate corresponding to Kendall’s tau

^b^ Correlation estimate corresponding to Pearson’s r

# **Table S5.** Raw data of Arctic cod, sculpin, and ringed seal age (years), length (cm), and axial girth (cm) measurements.

| **Sample ID** | **Species** | **Location** | **Age (years)** | **Length (cm)** | **Axial Girth (cm)** |
| --- | --- | --- | --- | --- | --- |
| W170032 | Arctic cod | Sanikiluaq | 6 | 29.5 | - |
| W170033 | Arctic cod | Sanikiluaq | 4 | 28 | - |
| W170034 | Arctic cod | Sanikiluaq | 5 | 32.7 | - |
| W170035 | Arctic cod | Sanikiluaq | 5 | 30.9 | - |
| W170036 | Arctic cod | Sanikiluaq | 5 | 27.2 | - |
| W170037 | Arctic cod | Sanikiluaq | 5 | 36 | - |
| W170038 | Arctic cod | Sanikiluaq | 5 | 43.4 | - |
| W170039 | Arctic cod | Sanikiluaq | 6 | 44.4 | - |
| W170040 | Arctic cod | Sanikiluaq | 5 | 39.8 | - |
| W170041 | Arctic cod | Sanikiluaq | 6 | 44.7 | - |
| W170042 | Arctic cod | Sanikiluaq | 8 | 54 | - |
| W170043 | Arctic cod | Sanikiluaq | 4 | 36.1 | - |
| W170044 | Arctic cod | Sanikiluaq | 4 | 41.3 | - |
| W170045 | Arctic cod | Sanikiluaq | 4 | 29.9 | - |
| W170046 | Arctic cod | Sanikiluaq | 5 | 44.4 | - |
| W170232 | Arctic cod | Kuujjuaraapik | 2 | 26 | - |
| W170233 | Arctic cod | Kuujjuaraapik | 3 | 32.5 | - |
| W170234 | Arctic cod | Kuujjuaraapik | 4 | 34.5 | - |
| W170235 | Arctic cod | Kuujjuaraapik | 2 | 25.5 | - |
| W170236 | Arctic cod | Kuujjuaraapik | 4 | 40 | - |
| W170237 | Arctic cod | Kuujjuaraapik | 3 | 25 | - |
| W170238 | Arctic cod | Kuujjuaraapik | 4 | 34 | - |
| W170239 | Arctic cod | Kuujjuaraapik | 4 | 35.5 | - |
| W170240 | Arctic cod | Kuujjuaraapik | 6 | 48.5 | - |
| W170241 | Arctic cod | Kuujjuaraapik | 5 | 49 | - |
| W170242 | Arctic cod | Kuujjuaraapik | 4 | 28.5 | - |
| W170243 | Arctic cod | Kuujjuaraapik | 5 | 44.5 | - |
| W170244 | Arctic cod | Kuujjuaraapik | 6 | 42.5 | - |
| W170245 | Arctic cod | Kuujjuaraapik | 4 | 35 | - |
| W170246 | Arctic cod | Kuujjuaraapik | 5 | 32 | - |
| W172221 | Arctic cod | Inukjuak | 3 | 30.5 | - |
| W172222 | Arctic cod | Inukjuak | 6 | 35.5 | - |
| W172223 | Arctic cod | Inukjuak | 5 | 33 | - |
| W172224 | Arctic cod | Inukjuak | 4 | 32.5 | - |
| W172225 | Arctic cod | Inukjuak | 8 | 45.5 | - |
| W172226 | Arctic cod | Inukjuak | 3 | 29 | - |
| W172227 | Arctic cod | Inukjuak | 4 | 27.5 | - |
| W172228 | Arctic cod | Inukjuak | 7 | 49.5 | - |
| W172229 | Arctic cod | Inukjuak | 5 | 32.5 | - |
| W172230 | Arctic cod | Inukjuak | 6 | 35.5 | - |
| W172231 | Arctic cod | Inukjuak | 3 | 23.5 | - |
| W172232 | Arctic cod | Inukjuak | 4 | 32 | - |
| W172233 | Arctic cod | Inukjuak | 4 | 29.5 | - |
| W172234 | Arctic cod | Inukjuak | 4 | 31.5 | - |
| W172295 | Arctic cod | Inukjuak | 3 | 21.5 | - |
| W180065 | Arctic cod | Inukjuak | 4 | 37.5 | - |
| W180066 | Arctic cod | Inukjuak | 3 | 34 | - |
| W180067 | Arctic cod | Inukjuak | 6 | 36 | - |
| W180068 | Arctic cod | Inukjuak | 4 | 27.5 | - |
| W180069 | Arctic cod | Inukjuak | 3 | 24.5 | - |
| W180070 | Arctic cod | Inukjuak | 3 | 26 | - |
| W180071 | Arctic cod | Inukjuak | 4 | 30.5 | - |
| W180072 | Arctic cod | Inukjuak | 3 | 28.5 | - |
| W180073 | Arctic cod | Inukjuak | 3 | 27 | - |
| W180074 | Arctic cod | Inukjuak | 3 | 24.5 | - |
| W180075 | Arctic cod | Inukjuak | 4 | 31 | - |
| W180076 | Arctic cod | Inukjuak | 7 | 37.5 | - |
| W170022 | Sculpin | Sanikiluaq | 4 | 25.3 | - |
| W170023 | Sculpin | Sanikiluaq | 6 | 22.5 | - |
| W170024 | Sculpin | Sanikiluaq | 3 | 21.8 | - |
| W170025 | Sculpin | Sanikiluaq | 6 | 27.5 | - |
| W170026 | Sculpin | Sanikiluaq | 4 | 23 | - |
| W170027 | Sculpin | Sanikiluaq | 3 | 22.2 | - |
| W170028 | Sculpin | Sanikiluaq | 4 | 28 | - |
| W170029 | Sculpin | Sanikiluaq | 4 | 23.8 | - |
| W170030 | Sculpin | Sanikiluaq | 4 | 25.5 | - |
| W170031 | Sculpin | Sanikiluaq | 3 | 23 | - |
| W170222 | Sculpin | Kuujjuaraapik | 3 | 23.5 | - |
| W170223 | Sculpin | Kuujjuaraapik | 2 | 24.5 | - |
| W170224 | Sculpin | Kuujjuaraapik | 4 | 26 | - |
| W170225 | Sculpin | Kuujjuaraapik | 6 | 26.5 | - |
| W170226 | Sculpin | Kuujjuaraapik | 6 | 19.4 | - |
| W170227 | Sculpin | Kuujjuaraapik | 4 | 24 | - |
| W170228 | Sculpin | Kuujjuaraapik | 3 | 24.5 | - |
| W170229 | Sculpin | Kuujjuaraapik | 6 | 27.5 | - |
| W170230 | Sculpin | Kuujjuaraapik | 5 | 28.5 | - |
| W172211 | Sculpin | Inukjuak | 8 | 29.5 | - |
| W172212 | Sculpin | Inukjuak | 6 | 27.5 | - |
| W172213 | Sculpin | Inukjuak | 7 | 37.5 | - |
| W172214 | Sculpin | Inukjuak | 5 | 24 | - |
| W172215 | Sculpin | Inukjuak | 6 | 26.5 | - |
| W172216 | Sculpin | Inukjuak | 5 | 25.5 | - |
| W172217 | Sculpin | Inukjuak | 5 | 26 | - |
| W172218 | Sculpin | Inukjuak | 4 | 24.5 | - |
| W172219 | Sculpin | Inukjuak | 10 | 33 | - |
| W172220 | Sculpin | Inukjuak | 4 | 24.5 | - |
| W180064 | Sculpin | Inukjuak | 8 | 28.5 | - |
| K14-37078 | Ringed seal | Kuujjuaraapik | - | 106.7 | 76.2 |
| K14-37079 | Ringed seal | Kuujjuaraapik | - | 142.2 | 104.1 |
| K14-37080 | Ringed seal | Kuujjuaraapik | - | 96.5 | 78.7 |
| K14-37081 | Ringed seal | Kuujjuaraapik | - | 147.3 | 108.0 |
| K14-39228 | Ringed seal | Kuujjuaraapik | - | 127.0 | 101.6 |
| K14-39229 | Ringed seal | Kuujjuaraapik | - | 144.8 | 108.0 |
| K14-39230 | Ringed seal | Kuujjuaraapik | - | 86.4 | 61.0 |
| K14-39231 | Ringed seal | Kuujjuaraapik | - | 123.8 | 110.5 |
| K14-39232 | Ringed seal | Kuujjuaraapik | - | 124.5 | 105.4 |
| K14-39234 | Ringed seal | Kuujjuaraapik | - | 137.2 | 113.0 |
| K14-39235 | Ringed seal | Kuujjuaraapik | - | 94.0 | 88.9 |
| K14-39236 | Ringed seal | Kuujjuaraapik | - | 130.5 | 102.6 |
| K14-39237 | Ringed seal | Kuujjuaraapik | - | 91.4 | 78.7 |
| K14-39238 | Ringed seal | Kuujjuaraapik | - | 100.6 | 76.5 |
| K16-43104 | Ringed seal | Sanikiluaq | - | 139.7 | 91.4 |
| K16-43105 | Ringed seal | Sanikiluaq | - | 121.9 | 78.7 |
| K16-43106 | Ringed seal | Sanikiluaq | - | 127.0 | 90.2 |
| K16-43107 | Ringed seal | Sanikiluaq | - | 154.9 | 92.7 |
| K16-43108 | Ringed seal | Sanikiluaq | - | 124.5 | 88.9 |
| K16-43109 | Ringed seal | Sanikiluaq | - | 139.7 | 96.5 |
| K16-43110 | Ringed seal | Sanikiluaq | - | 109.9 | 67.3 |
| K16-43303 | Ringed seal | Kuujjuaraapik | - | 139.7 | 99.1 |
| K16-43304 | Ringed seal | Kuujjuaraapik | - | 107.3 | 75.6 |
| K16-43305 | Ringed seal | Kuujjuaraapik | - | 127.0 | 108.0 |
| K16-43306 | Ringed seal | Kuujjuaraapik | - | 106.7 | 85.1 |
| K16-43307 | Ringed seal | Kuujjuaraapik | - | 157.5 | 114.3 |
| K16-43308 | Ringed seal | Kuujjuaraapik | - | 162.6 | 114.3 |
| K16-43309 | Ringed seal | Kuujjuaraapik | - | 149.9 | 109.2 |
| K16-43310 | Ringed seal | Kuujjuaraapik | - | 147.3 | 116.8 |
| K16-43314 | Ringed seal | Kuujjuaraapik | - | 86.4 | 55.9 |
| W172098 | Ringed seal | Sanikiluaq | - | 91.4 | 63.5 |
| W172099 | Ringed seal | Sanikiluaq | - | 78.7 | 63.5 |
| W172100 | Ringed seal | Sanikiluaq | - | 78.7 | 58.4 |
| W172101 | Ringed seal | Sanikiluaq | - | 152.4 | 132.1 |
| W172102 | Ringed seal | Sanikiluaq | - | 119.4 | 88.9 |
| W172103 | Ringed seal | Sanikiluaq | - | 106.7 | 68.6 |
| W172104 | Ringed seal | Sanikiluaq | - | 91.4 | 63.5 |
| W172105 | Ringed seal | Sanikiluaq | - | 96.5 | 71.1 |

**Table S6.** Results from within-species Kendall and Pearson correlations between metal concentrations (µg/g dw), in muscle and in liver. Liver concentrations were lipid normalized. Significant results bolded.

|  | | **Cadmium** | | **Mercury** | |
| --- | --- | --- | --- | --- | --- |
| Species (Common name) | n | r | P-value | r | P-value |
| Common eider | 27 | 0.52^b^ | **0.006** | 0.789^b^ | **<0.001** |
| Ringed seal | 29 | 0.59^a^ | **<0.001** | 0.834^b^ | **<0.001** |

^a^ Correlation estimate corresponding to Kendall’s tau

^b^ Correlation estimate corresponding to Pearson’s r

# **Table S7.** Average (± SD) of nitrogen, carbon and sulfur stable isotope values of species sampled from east Hudson Bay by location

| **Species** | **Location** | **n** | **δN15 ± SD** | **δC13 ± SD** | **δS34 ± SD** |
| --- | --- | --- | --- | --- | --- |
| Arctic cod | Inukjuak | 27 | 14.6 ± 1.0 | -19.0 ± 1.1 | 18.6 ± 1.5 |
|  | Kuujjuaraapik | 15 | 14.0 ± 0.9 | -20.9 ± 0.5 | 19.9 ± 1.00 |
|  | Sanikiluaq | 15 | 15.7 ± 1.1 | -18.3 ± 1.1 | 18.1 ± 1.4 |
| Blue mussel | Inukjuak | 16 | 8.0 ± 0.5 | -22.4 ± 0.6 | 19.1 ± 0.6 |
|  | Kuujjuaraapik | 10 | 6.2 ± 0.4 | -23.0 ± 0.3 | 20.7 ± 1.5 |
|  | Sanikiluaq | 20 | 8.0 ± 0.6 | -22.7 ± 0.6 | 19.4 ± 2.2 |
|  | Umiujaq | 6 | 7.6 ± 0.6 | -23.4 ± 0.5 | 19.2 ± 0.9 |
| Common eider | Inukjuak | 4 | 11.2 ± 0.3 | -20.3 ± 0.2 | 13.0 ± 0.6 |
|  | Kuujjuaraapik | 24 | 10.3 ± 0.9 | -19.1 ± 1.0 | 15.8 ± 2.9 |
|  | Sanikiluaq | 16 | 11.0 ± 0.4 | -19.6 ± 0.5 | 15.6 ± 2.8 |
|  | Umiujaq | 16 | 10.9 ± 0.8 | -19.2 ± 1.0 | 16.1 ± 1.8 |
| Ringed seal | Kuujjuaraapik | 23 | 14.9 ± 0.7 | -20.8 ± 0.6 | 17.3 ± 0.6 |
|  | Sanikiluaq | 14 | 14.5 ± 1.3 | -21.1 ± 1.7 | 16.1 ± 1.2 |
| Sculpin | Inukjuak | 11 | 14.0 ± 1.7 | -15.7 ± 3.1 | 15.5 ± 3.0 |
|  | Kuujjuaraapik | 10 | 14.7 ± 0.5 | -20.5 ± 0.3 | 19.9 ± 1.1 |
|  | Sanikiluaq | 10 | 14.9 ± 0.8 | -18.1 ± 0.9 | 19.2 ± 1.2 |
| Sea urchin | Inukjuak | 4 | 7.0 ± 0.2 | -19.5 ± 0.6 | 18.4 ± 0.9 |
|  | Kuujjuaraapik | 5 | 6.0 ± 0.2 | -18.0 ± 0.5 | 19.0 ± 0.5 |
|  | Sanikiluaq | 10 | 7.4 ± 0.9 | -18.6 ± 1.0 | 19.4 ± 0.5 |

# **Table S8.** Summary of among-species GLMMs explaining differences in log-transformed MeHg-corrected concentrations (µg/g dw) in tissues of 6 species from east Hudson Bay. Liver concentrations were used for vertebrate species. Liver concentrations were lipid normalized. The explanatory variables included in the model were δ^15^N, δ^13^C, and δ^34^S. Location of sample collection was included as a random effect. Significant results are shown in bold.

| **Model** |  | **Estimate (β coef)** | **SE** | **df** | **t-value** | **P-value** | **Variance** | **SD** |
| --- | --- | --- | --- | --- | --- | --- | --- | --- |
| 1. **logMeHg ~ δ15N + δ13C + δ34S + (1 \| Location), n = 216** | | | | | | | | |
| Fixed effects | (Intercept) | -0.322 | 0.407 | 122.320 | -0.790 | 0.431 |  |  |
|  | δ^15^N | 0.093 | 0.011 | 209.928 | 8.829 | **<0.001** |  |  |
|  | δ^13^C | -0.053 | 0.017 | 209.596 | -3.096 | **0.002** |  |  |
|  | δ^34^S | -0.147 | 0.013 | 209.844 | -11.078 | **<0.001** |  |  |
| Random effects | Location |  |  |  |  |  | 0.070 | 0.265 |
|  | Residual |  |  |  |  |  | 0.206 | 0.454 |

# **Table S9.** Results from among-species Kendall’s correlation analyses between log Cd concentrations and stable-isotopes. Muscle was used for vertebrate species. Arctic cod and sculpin samples were not included due to >50% of Cd samples being below the detection limit. Significant results are shown in bold. n = 127

| **log Cd ~** | | | |
| --- | --- | --- | --- |
|  | **z** | **tau** | **p-value** |
| d15N | -6.5442 | -0.39 | **<0.001** |
| d13C | -6.2859 | -0.38 | **<0.001** |
| d34S | 6.9508 | 0.42 | **<0.001** |

# **Table S10.** Results from among-species Kendall’s correlation analyses between log Hg concentrations and stable-isotopes. Liver was used for vertebrate species. Significant results are shown in bold. n = 240

| **log Hg ~** | | | |
| --- | --- | --- | --- |
|  | **z** | **tau** | **p-value** |
| d15N | 8.3994 | 0.36 | **<0.001** |
| d13C | 0.8543 | 0.04 | 0.393 |
| d34S | -8.4188 | -0.37 | **<0.001** |

# **Table S11.** Average methylmercury concentrations in tissue and average stable isotope values by species and location for amphipod, copepod, and gut content samples. Metal concentrations are recorded as dry weight concentrations ± SD. Hyphen (-) indicates no value available.

| **Species** | **Location** | **Tissue** | **n** | **MeHg ± SD**  **(µg/g DW)** | **δN15 ± SD** | | **δC13 ± SD** | **δS34 ± SD** | |
| --- | --- | --- | --- | --- | --- | --- | --- | --- | --- |
| Pelagic amphipods | Kuujjuaraapik | Whole Body | 1 | 0.019 | 9.3 | | - 23.3 | | 18.4 |
|  |  |  |  |  |  |  |  |  |  |
| Pelagic copepods | Sanikiluaq | Whole Body | 1 | 0.007 | 10.6 | | -25.1 | | 18.2 |
| **Gut Contents** | | | | | |  |  |  |  |
| Amphipod | Inukjuak | Whole Body | 1 | 0.059 | 9.1 | | -19.1 | | 19.9 |
|  | Kuujjuaraapik | Whole Body | 1 | 0.085 | 7.6 | | -22.1 | | 18.7 |
|  | Sanikiluaq | Whole Body | 1 | 0.109 | 7.0 | | -18.3 | | 17.3 |
| Cumacean | Inukjuak | Whole Body | 1 | 0.040 | 8.5 | | -18.9 | | 18.3 |
| Prey fish | Inukjuak | Whole Body | 2 | 0.113 ± 0.110 | 13.8 ± 3.3 | | -20.6 ± 1.0 | | 16.6 ± 5.3 |
|  | Kuujjuaraapik | Whole Body | 1 | 0.103 | 10.2 | | -21.4 | | 18.5 |
|  | Sanikiluaq | Whole Body | 4 | 0.066 ± 0.049 | 12.4 ± 1.5 | | -19.9 ± 2.0 | | 17.4 ± 2.4 |
| Mysid | Inukjuak | Whole Body | 1 | 0.016 | 10.2 | | -21.7 | | - |
| Shrimp | Sanikiluaq | Whole Body | 1 | 0.040 | 11.1 | | -20.2 | | 18.6 |

# **Table S12.** Summary of among-species GLMMs explaining differences in log Hg concentrations (µg/g dw) in tissues of 12 species from east Hudson Bay. Muscle was used for vertebrates. The explanatory variables included in the model were δ15N, δ13C, and δ34S. Location of sample collection was included as a random effect. Significant results are shown in bold.

| **Model** |  | **Estimate (β coef)** | **SE** | **df** | **t-value** | **P-value** | **Variance** | **SD** |
| --- | --- | --- | --- | --- | --- | --- | --- | --- |
| 1. **logHg ~ δ15N + δ13C + δ34S + (1 \| Location), n = 248** | | | | | | | | |
| Fixed effects | (Intercept) | -1.436 | 0.245 | 102.160 | -5.856 | **<0.001** |  |  |
|  | δ^15^N | 0.144 | 0.006 | 242.135 | 24.122 | **<0.001** |  |  |
|  | δ^13^C | 0.008 | 0.010 | 241.987 | 0.781 | 0.436 |  |  |
|  | δ^34^S | -0.051 | 0.008 | 241.821 | -6.332 | **<0.001** |  |  |
| Random effects | Location |  |  |  |  |  | 0.032 | 0.180 |
|  | Residual |  |  |  |  |  | 0.077 | 0.277 |

# **Table S13.** Summary of trophic magnification slope (TMS) analysis for Hg biomagnification in an east Hudson Bay arctic marine food web. Dataset expanded to include gut content data in analysis. Muscle used for vertebrates. Significant results are shown in bold.

| **Model** |  | **Estimate (β coef)** | **SE** | **t-value** | **P-value** |
| --- | --- | --- | --- | --- | --- |
| 1. **logHg ~ δ15N** | | | | | |
| R^2^ = 0.65, n = 249, F-statistic = 456.7, P-value <0.001 | | | | | |
|  | (Intercept) | -2.577 | 0.085 | -30.16 | **<0.001** |
|  | δ15N | 0.149 | 0.007 | 21.37 | **<0.001** |


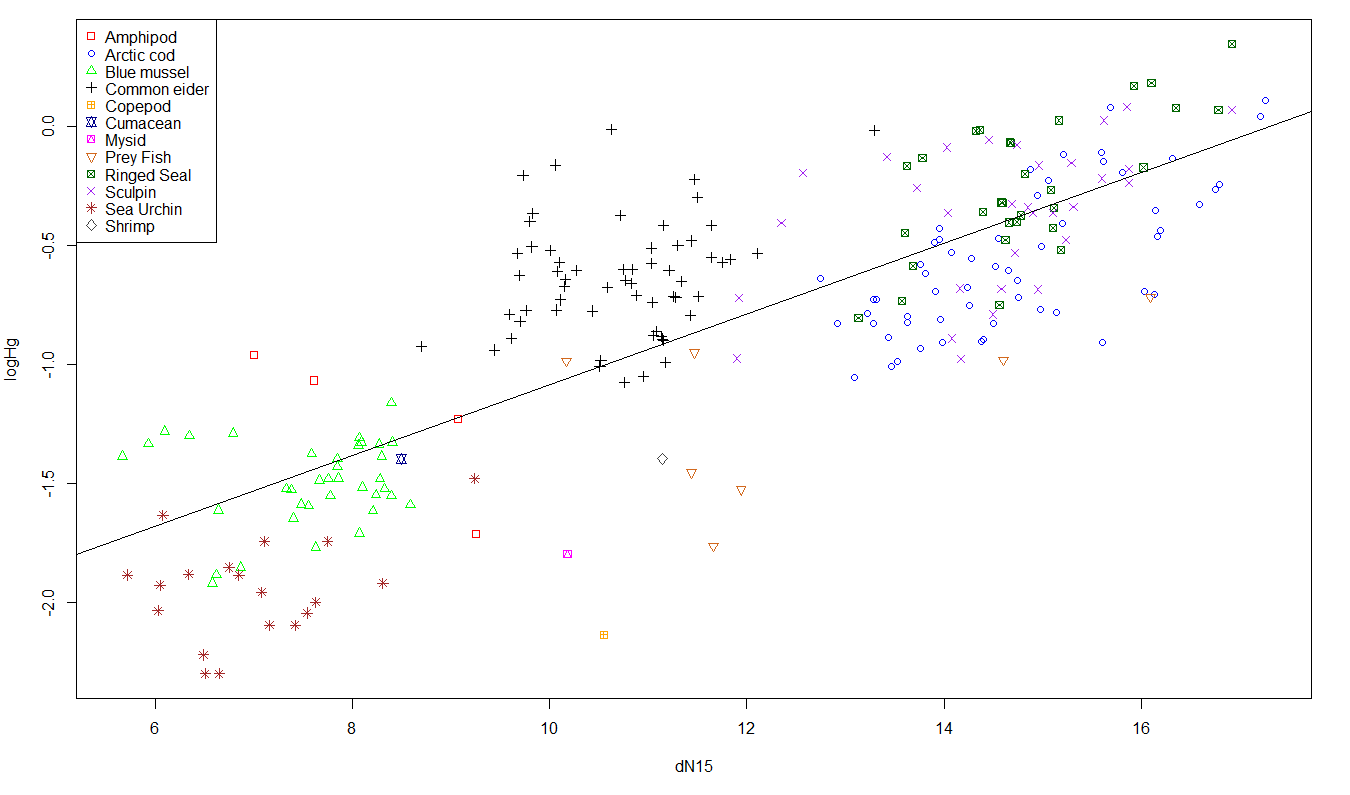


# **Fig. S1** Trophic magnification slope (TMS) of log Hg in east Hudson Bay food web. Using muscle for vertebrates. Dataset expanded to include gut content data in analysis.
